# Does *in vitro* fertilization (IVF) treatment provide good value for money? A cost-benefit analysis

**DOI:** 10.3389/fgwh.2023.971553

**Published:** 2023-03-01

**Authors:** Elena Keller, Willings Botha, Georgina M. Chambers

**Affiliations:** Centre for Big Data Research in Health & National Perinatal Epidemiology and Statistics Unit, UNSW Sydney, Sydney, NSW, Australia

**Keywords:** cost-benefit analysis (CBA), IVF treatment, value of a statistical baby (VSB), health technology assessment (HTA), Markov model, willingness-to-pay (WTP), discrete choice experiment (DCE)

## Abstract

**Background:**

Using traditional health technology assessment (HTA) outcome metrics, such as quality-adjusted life-years, to assess fertility treatments raises considerable methodological challenges because the objective of fertility treatments is to create new life rather than extend, save, or improve health-related quality of life.

**Objective:**

The aim of this study was to develop a novel cost-benefit framework to assess value for money of publicly funded IVF treatment; to determine the number of cost-beneficial treatment cycles for women of different ages; and to perform an incremental cost-benefit analysis from a taxpayer perspective.

**Methods:**

We developed a Markov model to determine the net monetary benefit (NMB) of IVF treatment by female age and number of cycles performed. IVF treatment outcomes were monetized using taxpayers' willingness-to-pay values derived from a discrete choice experiment (DCE). Using the current funding environment as the comparator, we performed an incremental analysis of only funding cost-beneficial cycles. Similar outputs to cost-effectiveness analyses were generated, including net-benefit acceptability curves and cost-benefit planes. We created an interactive online app to provide a detailed and transparent presentation of the results.

**Results:**

The results suggest that at least five publicly funded IVF cycles are cost-beneficial in women aged <42 years. Cost-benefit planes suggest a strong taxpayer preference for restricting funding to cost-beneficial cycles over current funding arrangements in Australia from an economic perspective.

**Conclusions:**

The provision of fertility treatment is valued highly by taxpayers. This novel cost-benefit method overcomes several challenges of conventional cost-effectiveness methods and provides an exemplar for incorporating DCE results into HTA. The results offer new evidence to inform discussions about treatment funding arrangements.

## 1. Introduction

Infertility affects one in eight couples (1) and >180 million people worldwide representing an increasing public health problem (2). The value of the global market for fertility treatments is estimated to reach US$27 billion in 2026 (3). The major contributory factors include a trend to later childbearing and, thus, an increase in age-related infertility, as well as an increase in the prevalence of infertility due to medical conditions such as obesity and reducing sperm counts (4–6).

### 1.1. Fertility treatments and public funding

Infertility causes significant personal suffering for couples, including long-term sadness, interruption to life plans, a sense of loss of meaning, and stigmatization (2, 7). Fertility treatments allow many of these couples to conceive. Various treatment options are available, of which *in vitro* fertilization (IVF) is most common with >2 million IVF cycles performed globally each year (8). At a global level, there is arguably no other medical intervention that displays such varying government and third-party funding arrangements as IVF (9), including significant variation in the eligibility criteria and maximum number of publicly financed treatment cycles (10–16). In the latest survey of assisted reproductive technology (ART) practices and policies undertaken in 2019 by the International Federation of Fertility Societies, less than half (47%) of reporting countries provided any type of financial support for ART. This compared to 64% in the previous survey conducted in 2016. Moreover, only 20% of reporting countries offered full reimbursement for ART services (17). For instance, there is no public funding for ART in the United States, although several states have insurance mandates for partial cover (18). In the United Kingdom, the National Institute for Health and Care Excellence recommends three full IVF cycles for women aged <40 years or one full cycle for women aged 40–42 years (19). While there is no limit on the number of IVF treatment cycles subsidized under the Australian universal health insurance scheme, called Medicare (20), only partial funding is available, meaning couples are still faced with substantial out-of-pocket costs.

### 1.2. Inadequacy of traditional health technology assessment methods

Arguably, the reason for such variation in funding arrangements is the lack of an adequate metric in economic evaluation that reflects the value that society places on fertility treatment (21). Although some research has been done on the comparative cost-effectiveness of various fertility treatments (22–28), there is no evidence on whether the provision of subsidized treatment provides good value for money.

Traditional health technology assessment (HTA) outcome metrics, which focus on health-related quality of life, such as cost per quality-adjusted life-years (QALYs) or disability-adjusted life-years (DALYs), are debatably inadequate when used for fertility treatments (21, 29, 30). Quality-adjusted life-years and DALYs were designed for, and thus are best suited to, measuring changes in health-related quality of life for already existing lives with utility weights derived from societal preferences for different health states (31). They are thus conceptually different to measuring the value gained through the future conception of a baby. Unlike most other types of medical care, fertility treatment is judged by its ability to create life rather than extend, save, or improve health-related quality of life. Therefore, the use of QALYs as outcome measure is limited to measuring the maternal and/or paternal health-related quality of life gains (e.g., prevention of psychological suffering resulting from involuntary childlessness). This only captures a component of the utility/disutility associated with fertility treatment, much of which is associated with non-health outcomes reflecting achieving life goals. Essentially, by undergoing fertility treatment individuals are buying hope to have a child and the potential to create a meaningful life, rather than improving health or avoiding disease (21, 30, 32, 33). The challenges of using QALYs to value fertility treatment are highlighted by a review of cost-effectiveness analyses for fertility treatments (34) that showed that only one of 14 studies employed QALYs. Contrastingly, using QALYs in the context of perinatal interventions (e.g., perinatal screening) is appropriate because the decision is about avoiding diseases in a life that has already been conceived (pregnancy or newborn) or is assumed to be conceived in the future (prenatal in prospective parents). Methodological challenges in economic evaluations of fertility treatments have been extensively highlighted in the literature (21, 29, 35). Cost-benefit analysis (CBA) which measures value in monetary units, is an alternative among existing HTA techniques that might be better equipped for an economic evaluation of therapies that create life, such as IVF treatment (29, 30), and potentially overcomes the challenge of incorporating non-health-related benefits. Cost-benefit analysis allows incorporation of willingness-to-pay (WTP) values to capture the value associated with a health intervention and holistically reflects the health and non-health preferences of relevant stakeholders.

### 1.3. Cost-benefit analysis framework

The basis of CBA methods is that goods and services – or fertility treatments in this case – should only be provided if doing so is profitable, that is the monetary value of the benefits exceeds the cost of achieving those benefits. In the context of IVF treatment, the decision-rule would be to provide treatment only when the benefits (e.g., a live birth) outweigh the costs of providing IVF, that is when the net monetary benefit (NMB) is positive. A taxpayer (general population) perspective was chosen because Australian taxpayers fund the majority of the cost of IVF through the universal health insurance scheme, Medicare, with no restriction on female age or number of cycles. Furthermore, the WTP for a statistical baby from an *ex-ante* taxpayer perspective would be less biased than from an *ex-post* patient perspective, with many patients willing to subject themselves to significant financial stress in the hope of having a child (36–38).

The success rates of fertility treatment generally decline with a woman's age (39–43), duration of infertility (41), and number of previously failed attempts (39). This implies that (1) expected costs of providing IVF treatment will outweigh expected benefits at some point and, therefore, suggests that there is a maximum number of cost-beneficial treatment cycles; (2) fertility treatment is less cost-beneficial in older compared to younger women, i.e., a smaller number of cycles would provide good value for money. Only two studies have undertaken a form of CBA for fertility treatment but did not account for age or number of cycles performed (44, 45).

Despite McIntosh (46) outlining hypothetically how to incorporate discrete choice experiment (DCE) results into a CBA framework and how to create outputs that are similar to cost-effectiveness analyses (CEAs) such as net-benefit acceptability curves, we are aware of only two studies that have attempted to incorporate DCE results into a CBA framework. One study compared WTP values for perinatal services with the costs (47), while another study (48) generated cost-benefit planes for a CBA alongside a randomized controlled trial in the context of dental primary care. The disconnect between the proliferation in DCEs and their minimal use in HTA is an area of growing research need (49–52).

### 1.4. Objectives

The aims of this study were to develop a CBA framework that uses DCE results to conduct an economic evaluation of IVF treatment and to determine the NMB stratified by female age and number of treatment cycles from a taxpayer perspective. An additional objective was to conduct an incremental CBA similar to traditional HTA methods using the current public funding arrangement in Australia as the comparator.

## 2. Materials and methods

### 2.1. Model structure

**Figure 1:**
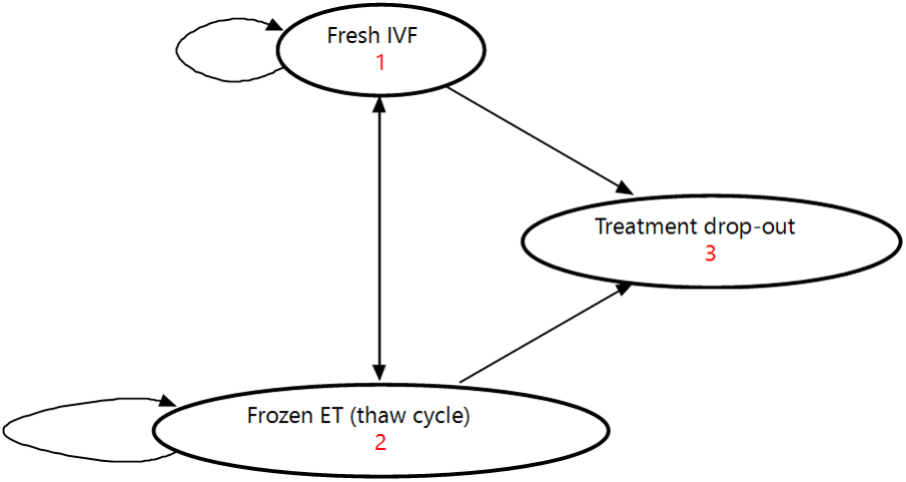
Illustration of Markov model used for analysis. ET, embryo transfer; IVF, *in vitro* fertilization.

A Markov decision analytic model (Figure 1) was constructed from a taxpayer (i.e., general population) perspective including all clinically and economically relevant stages of IVF treatment. This means the key outcomes possible from treatment initiation to live birth or unsuccessful treatment were represented. A fresh IVF cycle (Supplementary Figure S1, Appendix A) was represented by controlled ovarian stimulation (COS), followed by oocyte pick-up (OPU) procedure. Oocyte pick-up is followed by egg fertilization to create an embryo, and then a fresh embryo transfer or a freeze-all embryo cycle. A thaw cycle (Supplementary Figure S2, Appendix A) was represented by thawing of embryos, and frozen/thaw embryo transfer or failure of the thaw resulting in no embryos being viable. At each stage of the fresh or thaw cycle the cycle could be discontinued for medical or non-medical reasons. Both a fresh and thaw cycle could result in a live birth (singleton or multiple birth) or a “failed cycle” including no clinical pregnancy, miscarriage, or stillbirth. We defined a Markov cycle as one fresh or thaw cycle (i.e., a maximum of one embryo transfer procedure), and a complete cycle as all fresh and thaw cycles resulting from one OPU procedure.

We constructed ten age-specific models for the following female age groups: <30, 30–31, 32–33, 34–35, 36–37, 38–39, 40–41, 42–43, 44–45, and >45 years.

The treatment pathways were defined using Markov health states representing cumulative numbers and types of IVF cycles (including IVF with fresh and frozen embryo transfer). Correspondingly, the length of Markov cycles was defined as one IVF cycle. The model was restricted to a time horizon of 20 Markov cycles, a maximum of eight complete IVF cycles, and a maximum of four live births per woman, meaning the model was terminated once one or more of these conditions were met. This model time horizon was chosen for the following reasons: (1) Twenty Markov cycles would allow up to 20 embryo transfers (i.e., fresh and thaw cycles) and, thus, should not pose a significant restriction. Currently women undertaking ART treatment in Australia undergo two cycles, on average, with approximately 11% of women undergoing four or more fresh and/or thaw cycles (53). (2) Due to the comprehensive data required for the model and the small number of women undergoing more than eight complete IVF treatment cycles, we limited the time horizon to a maximum of eight complete cycles. In comparison, cost-effectiveness analyses of IVF treatment generally only include up to 3–4 cycles (34). (3) The number of live births was restricted to a maximum of four given that it is highly unlikely women will undergo further fertility treatment and given that, currently, 99% of families with children have ≤4 children (54). No discounting was applied because the perspective of the analysis is the costs incurred and the benefits obtained over a one-year period, hence discounting was inconsequential. Permanent treatment drop-out (meaning women do not return for further IVF cycles) was defined as the absorbing state. The outcome was defined as a live birth of at least one child, which was subsequently monetized for the CBA.

### 2.2. Utilization of IVF treatment

We populated the model with data on initiated autologous IVF cycles undertaken in Australia between 2014 and 2018 (i.e., the most recent years with available data). We only included data for women commencing IVF treatment between 2014 and 2016 to allow for sufficient follow-up time and undergoing multiple cycles. We excluded data for cycles with <30 women per complete cycle and age group. In affected age groups (44–45 and >45-year-old women) we only analyzed the model up to the last complete cycle with data for ≥30 women (up to the seventh complete cycle in 44–45-year-old women and the third complete cycle in >45-year-old women). The proportion of women reaching each stage of the model was applied to a population of 100,000 women in each age group during model analysis.

### 2.3. Data sources

#### 2.3.1. Costs

Given the taxpayer (general population) perspective specified by the DCE, we included direct healthcare costs funded through Australia's national health insurance scheme, Medicare. Information on treatment costs was obtained from previous analyses of fertility treatment costs (55) together with a survey of tariffs charged by Australian fertility clinics. Direct costs comprised Medicare Benefits Schedule and Pharmaceutical Benefits Scheme expenditure. In line with the DCE, patient out-of-pocket payments were not considered in the model. In addition, we included indirect costs resulting from increased health risks of multiple births compared to singleton infants (55). These indirect costs were measured in terms of the government-funded hospital costs for the infants in their first year of life. All costs are expressed in undiscounted 2020 Australian dollars [2020 purchasing power parity: AU$1 = US$0.71 (56)].

To derive the average costs per taxpayer for each stage of treatment within the model (i.e., each node in Supplementary Figures S1 and S2, Appendix A), we divided each cost item (e.g., government rebate for COS) by 320.25, the average number of taxpayers funding each complete fertility treatment cycle in Australia (14,288,292 taxpayers in financial year 2017–2018 (57) divided by 44,616 complete fertility treatment cycles conducted in 2018 in Australia (58)). This approach allowed us to explore the impact of (1) the number of taxpayers as well as (2) the number of complete fertility treatment cycles conducted annually in Australia on the CBA results in sensitivity analyses.

#### 2.3.2. Live births

Transition probabilities, which include treatment discontinuation and success rates, were sourced from the Australian and New Zealand Assisted Reproduction Database (ANZARD). ANZARD is a clinical registry of IVF cycles performed by all Australian fertility clinics and complete ascertainment is assumed (58). Data from the ANZARD 2014–2018 Australian cohort provided live-birth rates by age group for women with successive IVF cycles which was critical for informing the CBA. Consistent with previous literature (22), we defined a live birth as the birth of at least one live-born baby >20 weeks of gestational age and surviving for ≥28 days. Furthermore, we counted live births as birth events, meaning a multiple live birth was counted as a single live-birth event.

#### 2.3.3. Monetary value of a baby

The WTP per taxpayer for a live birth can be defined similarly to the value of a statistical life as the WTP for a marginal increase in the chance of having a baby – also called the value of a statistical baby (VSB) (30). The literature on VSB estimates is limited with only one study from 1994 reporting estimates as part of a feasibility study (59). Instead, to derive the VSB, we used WTP values for fertility treatment attributes that were elicited using a stated-preference DCE among Australian taxpayers (60, 61). In this DCE, participants were asked to choose between fertility treatment programs described by seven attributes/characteristics, thereby trading off additional annual tax payments against higher treatment success rates among other characteristics. The taxpayer perspective was chosen because it reflects the healthcare payer HTA perspective. More details regarding the design and analysis of the DCE can be found in Supplementary Appendix B. Botha, Donnolley (61) estimated taxpayer's WTP for a 1% increase in the treatment live-birth rate to be $2.23 in additional tax contributions, meaning the taxpayer VSB would be $223 in additional tax contributions, which captures taxpayers' valuation for parental health and non-health benefits as well as for the potential to form a family and achieve life goals. Similar to the value of a statistical life, this VSB represents the WTP for a marginal increase in the treatment live-birth rate, rather than the WTP for an identified life.

### 2.4. Model analysis

Given that the probability of a live birth after fertility treatment in women using their own eggs depends predominately on female age, and the number of available embryos for transfer (39, 40, 62), we used Monte Carlo simulation (i.e., microsimulation), instead of a cohort analysis, to analyze the model. Microsimulation tracks single patients with specific characteristics through the Markov model, thereby determining their outcomes and associated costs. Numerous patients (base-case: n = 100,000 per age group) were simulated and the average cost and benefit values for these patients have then been compared.

To calculate the average costs per taxpayer per complete IVF cycle by age, we multiplied the proportion of women reaching each stage of the Markov model with the corresponding average cost per taxpayer for the different types of partial and complete cycles of IVF treatment and summed all costs by age group and cycle.

We then calculated the average age- and cycle-specific cost per taxpayer and live birth as the average costs per taxpayer per complete cycle by age divided by the age- and cycle-specific live-birth rate. The main outcome measure of the CBA was the NMB for each age group and cycle [Equation (1)]. It was calculated as the WTP per taxpayer for a live birth (VSB) minus the average age- and cycle-specific cost per taxpayer and live birth.

**Equation 1:** Calculation of the net monetary benefit (NMB).


(1) $$\begin{array}{rl} \mathrm{NMB}= & \mathrm{Benefits}\;(\text{WTP per taxpayer for live birth})\\ & -\mathrm{Costs}\;\left(\mathrm{average cost per taxpayer for live birth}\right) \end{array}$$

We performed all analyses from two perspectives: (1) We only considered women in each complete cycle who reached OPU in the Markov model, meaning that all women dropping out of treatment prior to undergoing OPU and their associated costs were excluded from the analysis. This scenario is often considered by decision-makers, reflects funding limits based on the number of OPUs, and was our primary analysis. (2) We considered all women initiating a treatment cycle (i.e., undergoing COS), meaning that all 100,000 patients simulated as well as their costs were considered. This scenario is a better reflection of the funding required for the provision of IVF as it also accounts for sunk costs attributed to women dropping out of treatment prior to OPU. This scenario reflects funding limits based on the number of initiated cycles.

We determined the number of treatment cycles that provide good value for money according to taxpayer preferences as the number of cycles with a positive NMB.

### 2.5. Sensitivity analyses

#### 2.5.1. One-way sensitivity analyses

We explored the uncertainty around the VSB, the number of fertility treatment cycles conducted annually, and the number of taxpayers in one-way sensitivity analyses (Table 1) as these are the input parameters assumed to be most uncertain and, hence, to potentially have the biggest impact on results. The number of fertility treatment cycles conducted annually and the number of taxpayers are relevant parameters as they jointly determine the average cost per taxpayer for a treatment cycle which is then compared to the taxpayer VSB. In other words, the marginal value per extra baby per taxpayer is constant, but the average cost per taxpayer per cycle is determined by the number of cycles performed and the number of taxpayers in a country. For instance, average costs per treatment cycle per taxpayer are reduced by half if the number of taxpayers in a country is held constant but the number of fertility treatment cycles performed is halved, and this would lead to a more favorable comparison against the taxpayer VSB and, thus, a higher NMB. Results were visualized using tornado plots. In addition, we conducted threshold analyses: Holding all other parameters (including treatment costs and transition probabilities) fixed, we calculated the threshold (1) VSB, (2) number of complete fertility treatment cycles conducted annually, and (3) number of taxpayers in Australia beyond which treatment in each age group and cycle does not provide good value for money.

**Table 1:** Input parameters for the cost-benefit analysis of IVF treatment from a taxpayer perspective in Australia.

Parameter	Base-case value	Range explored in one- and/or two-way sensitivity analyses	Distribution used for probabilistic sensitivity analyses	Source
WTP for a baby	$223	$164 – $282	Normal (SD = 29.5)	(61)
Number of fertility treatment cycles conducted annually^1^	44,616	10,000–100,000	Multinormal (SD = 5,000; correlation with number of taxpayers = 0.91)	(58)
Number of taxpayers	14,288,292	10,000,000–20,000,000	Multinormal (SD = 1,000,000; correlation with number of fertility treatment cycles conducted annually = 0.91)	(57)
MBS and PBS costs		NA	Gamma (±20%)	Survey of tariffs charged by fertility clinics; MBS and PBS
Hospital costs for birth admission up to one year of life		NA	Gamma (±40%)	(55)
Transition probabilities		NA	Beta (±5%)	ANZARD data

1 The number of fertility treatment cycles conducted annually includes all complete autologous, oocyte donation and GIFT (gamete intrafallopian transfer) cycles conducted in Australia. ANZARD, Australian and New Zealand Assisted Reproduction Database; MBS, Medicare Benefits Schedule; PBS, Pharmaceutical Benefits Scheme; SD, standard deviation; WTP, willingness-to-pay.

#### 2.5.2. Two-way sensitivity analyses

The number of taxpayers and the number of fertility treatment cycles conducted annually in Australia historically had a strong positive correlation with a correlation coefficient of 91.21% based on data from 2005 to 2018 (57, 58, 63–88). Consequently, with an increase in the number of taxpayers, which likely means an increase in the number of reproductive-aged women, the number of fertility treatment cycles conducted annually tends to go up and they jointly determine the average costs of fertility treatment provision per taxpayer. In two-way sensitivity analyses we determined the number of cost-beneficial cycles by age for each combination of the number of taxpayers (range: 10–20 million) and fertility treatment cycles conducted annually (range: 10,000–100,000). Results were presented using area charts.

#### 2.5.3. Probabilistic sensitivity analyses (PSA)

In PSA a probability distribution was used to quantify the uncertainty around relevant parameters (Table 1). We also used a correlation coefficient of 91.21% between the annual number of fertility treatment cycles and the number of taxpayers to define a multinormal distribution. To conduct the PSA, we performed 1,000 iterations of simultaneously simulating 1,000 women in the model. The results were used to construct net-benefit acceptability curves which visualize the probability of a scenario providing good value for money (46) and to estimate 95% confidence intervals (CI) around the base-case results using a percentile approach (89).

### 2.6. Incremental analyses

To support the results of the base-case and sensitivity analyses, we conducted an incremental analysis using the current funding environment as the comparator (additional details in Supplementary Appendix C). This allowed us to construct incremental cost-benefit ratios (ICBR) plotted in a cost-benefit plane following the approach outlined in McIntosh (46). We defined the comparator as funding the current “package” of fertility treatment in Australia, which is characterized by providing funding for an unlimited number of cycles in all age groups. To facilitate the analysis, we derived the costs and benefits per taxpayer of an average cycle in this package according to Equation (2). Using utilization data for 2018 (based on ANZARD) to determine the proportion of cycles stratified by age group and complete cycle, we calculated the sum of proportionate costs and benefits per taxpayer in each cycle and age group. For instance, if a first complete cycle in <30-year-old women costs $30 per taxpayer and 1% of all cycles conducted annually represent first complete cycles in this age group, then we would consider $0.30 ($30 × 1%) in the calculation for the costs of an average cycle in the current package. The other summands are calculated equivalently and represent the costs in all other cycles and age groups. The calculation for the benefits of an average cycle in the current package is equivalent.

**Equation 2:** Calculation of the costs per taxpayer for an average cycle in the current package of fertility treatment.


(2) $$\begin{array}{rl} & \mathrm{Costs}\;\;\mathrm{per}\;\mathrm{taxpayer}\;\mathrm{of}\;\mathrm{average}\;\mathrm{cycle}\;\mathrm{in}\;\mathrm{current}\;\;\mathrm{package}\\ & =\sum_{\mathrm{Age}\;\mathrm{Group}=<30}^{\mathrm{Age}\;\mathrm{Group}=>45}\sum_{\mathrm{Complete}\;\mathrm{Cycle}=1}^{\mathrm{Complete}\;\mathrm{Cycle}=8}\\ & \frac{\mathrm{No}.\;\mathrm{cycle}s_{\mathrm{Age}\;\mathrm{Group},\;\mathrm{Complete}\;\mathrm{Cycle}}}{\sum \sum \mathrm{No}.\;\mathrm{cycle}s_{\mathrm{Age}\;\mathrm{Group},\;\mathrm{Complete}\;\mathrm{Cycle}}}*\mathrm{Cost}s_{\mathrm{Age}\;\mathrm{Group},\;\mathrm{Complete}\;\mathrm{Cycle}} \end{array}$$

The “policy intervention” which we compared to the current funding arrangement was defined as funding the number of cycles that were deemed cost-beneficial stratified by age in the CBA. To determine the sum of age- and cycle-specific proportionate costs and benefits per taxpayer in the policy intervention we used the same utilization data. Prior to determining the proportion of cycles stratified by age group and complete cycle, we excluded any complete cycles that were not found to provide good value. Otherwise, calculations were equivalent.

Finally, we determined incremental costs and incremental benefits as the difference in average costs and benefits per taxpayer, respectively, between the policy intervention and the current package of fertility treatment.

### 2.7. Cost-effectiveness analysis

For comparison with economic evaluations of fertility treatment, including IVF treatment, reported in the literature, we performed an additional cost-effectiveness analysis of IVF treatment based on the same Markov model structure as for the CBA. Outcomes are reported as (1) cost per live birth, and (2) cost per QALY gained. Detailed methodology and results are provided in Supplementary Appendix D.

### 2.8. Online app

In addition to results presented within the manuscript and appendix, we created an interactive online app [https://elenakeller.shinyapps.io/CBA_IVF_Treatment/] with additional tables and figures, including:

• Summary statistics by age group and complete cycle for base-case analyses
• Tornado plots and threshold graphs for one-way sensitivity analyses
• Area charts for two-way sensitivity analyses
• Net-benefit acceptability curves for PSA
• Incremental cost-benefit planes for incremental analyses

This allowed us to provide our findings in a transparent, accessible way, and has several advantages over providing tables and figures as part of the appendix: (1) Given the complexity of our CBA, we generated >250 figures. The online app allows readers to choose the scenario (per OPU or per initiated cycle), age group, and complete cycle (e.g., first complete cycle) for which they would like to see the corresponding results. By changing the scenario, age group, and/or complete cycle, readers can directly observe the impact of these changes on the outcome measures and can more easily determine general trends. (2) Outcome measures such as the NMB varied substantially between age groups, particularly between women aged <42 and ≥42 years. If plotting all age groups in one figure, data from younger age groups is compressed to the degree that differences between age groups become indiscernible. In contrast, in the online app, axes can be adjusted using a slider to select the range of interest. (3) Responsive labels for single data points in figures provide exact values of outcome measures, whereas in an appendix this level of detail would not be possible.

Examples of all tables and figures are provided in Supplementary Appendix E for the 38–39-year-old age group.

Ethics approval was obtained from the University of New South Wales, Sydney (HC16983). The study has been performed in accordance with the ethical standards of the Declaration of Helsinki. All analyses were performed using TreeAge software version 2021, R1 (90) and R version 4.0.3 (91).

## 3. Results

### 3.1. Base-case analyses

**Figure 2:**
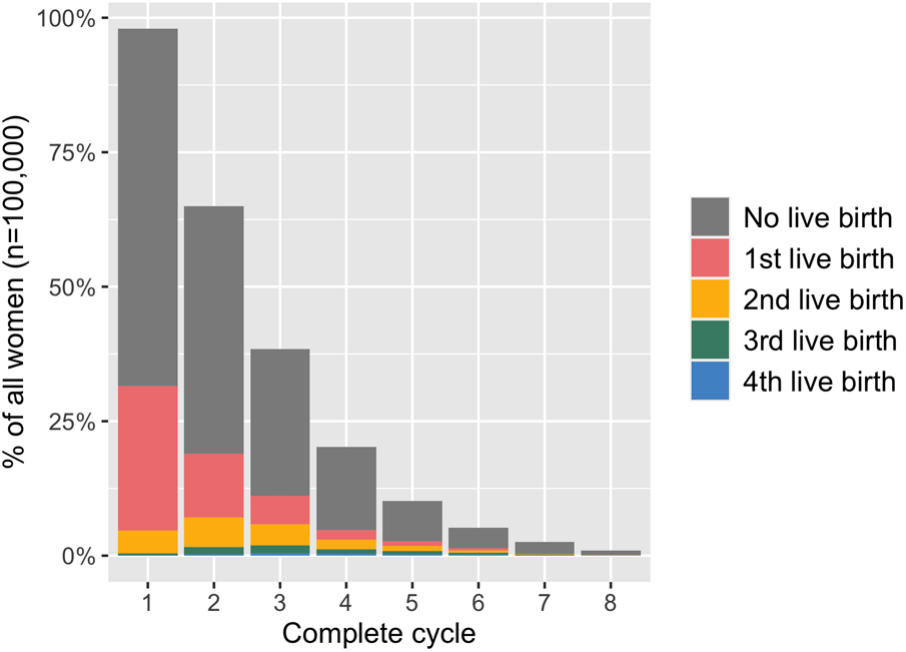
Treatment outcomes in 38-39-year-old women stratified by complete cycle. The height of the bar indicates the proportion of all women (*n* = 100,000) who started each complete cycle (as defined by undergoing an oocyte pick-up procedure).

Average live-birth rates per woman after up to eight complete cycles as well as the number of women with one, two, three and four live births in total are displayed by age group in Table 2. Additionally, Figure 2 illustrates treatment outcomes stratified by complete cycle for 38–39-year-old women as an example. Due to high treatment drop-out rates in each cycle <1% of women proceeded to an eighth complete cycle in this age group.

**Table 2:** Cumulative live-birth rates and number of women with live births by age group for the base-case analysis.

Age group	No. live births	Average live-birth rate per woman after the 8th complete cycle	No. women with a 1st live birth	No. women with a 2nd live birth	No. women with a 3rd live birth	No. women with a 4th live birth
<30 years	140,626	1.41	71,909	40,097	19,757	8,863
30–31 years	148,112	1.48	73,646	42,746	21,687	10,033
32–33 years	140,337	1.40	71,828	40,344	19,631	8,534
34–35 years	116,066	1.16	64,513	31,921	14,029	5,603
36–37 years	99,561	1.00	58,634	26,299	10,700	3,928
38–39 years	70,689	0.71	47,109	16,811	5,225	1,544
40–41 years	44,267	0.44	33,769	8,353	1,774	371
42–43 years	17,332	0.17	15,541	1,629	150	12
44–45 years	6,004	0.06	5,759	239	6	0
>45 years	1,561	0.02	1,547	14	0	0

Average live-birth rate per woman after the 8th complete cycle is calculated as the number of live births divided by the number of women per age group simulated in the Markov model (*n* = 100,000). No., number.

**Figure 3:**
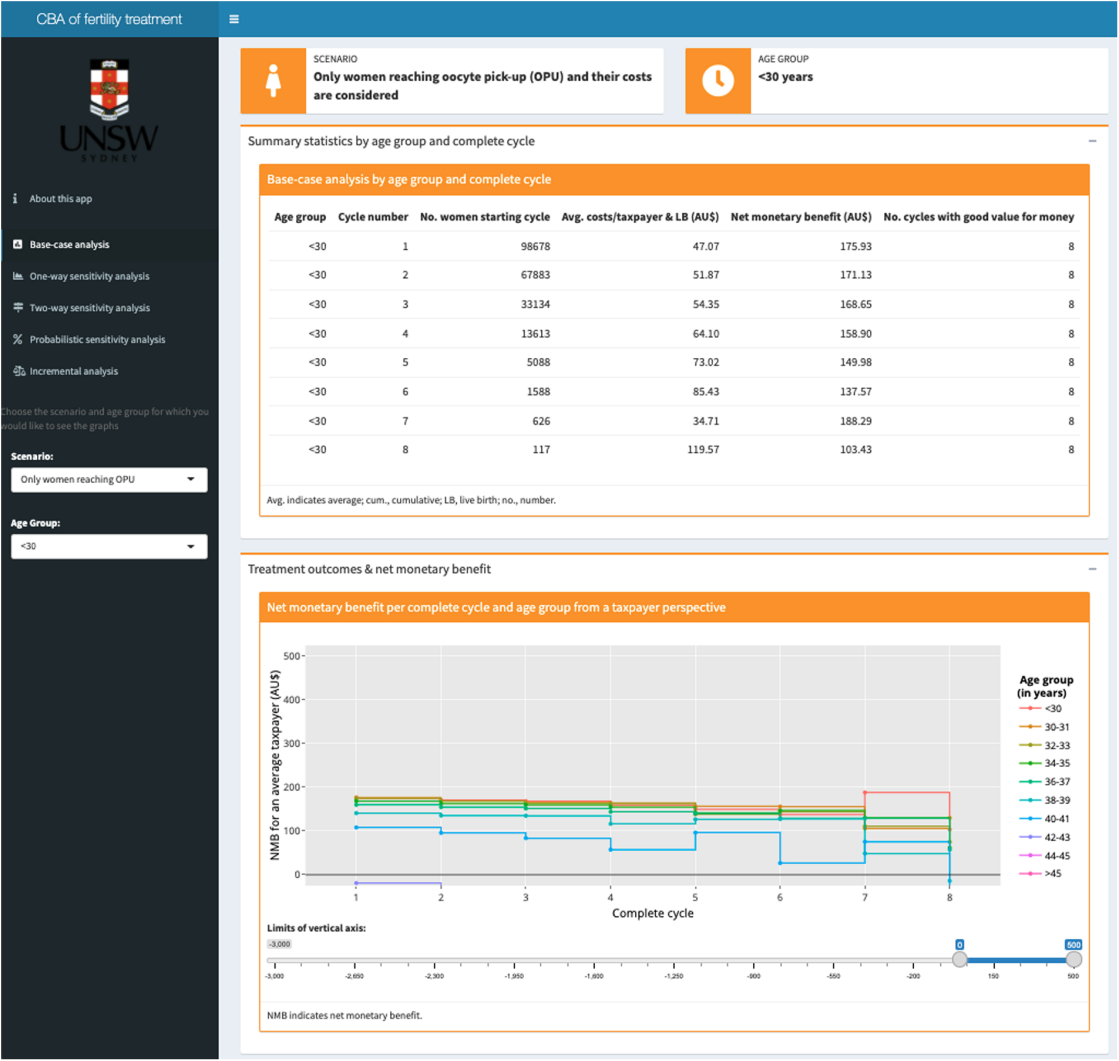
Screenshot of the base-case analysis results page in the interactive online app showing (1) a table with summary statistics at the top; and (2) a graph plotting the net monetary benefit across all cycles by age group at the bottom. Based on the selection in the dark grey panel on the left for the (1) scenario (per OPU or per initiated cycle) and (2) age group, tables and figures are automatically updated.

Results for both scenarios considered (per OPU and per initiated cycle) were similar, hence, we only report on the main analysis (i.e., per OPU). Table 3 provides base-case results for 38–39-year-old women as an example. We refer readers to the online app for additional tables and figures (Figure 3). The example results show the decrease in the number of women starting a cycle (from 97,953 to 1,011) as well as the decreasing trend in the cycle-specific live-birth rate over successive cycles (from 0.32 to 0.09 live births per cycle). Generally, the lower the cycle-specific live-birth rate, the higher the average costs per taxpayer and live birth, and the smaller the NMB. In 38–39-year-old women, the NMB tended to decrease over successive cycles and it turned negative in the eighth complete cycle, meaning seven complete IVF cycles were cost-beneficial.

**Table 3:** Summary statistics for the base-case analysis of the cost-benefit analysis in 38–39-year-old women in the scenario where only women reaching oocyte pick-up and their costs were considered.

Complete cycle	No. women starting cycle	No. LBs	Cycle-specific LB rate (95% CI)	Cum. no. LBs	Cum. LB rate (95% CI)	Avg. costs per taxpayer for a LB (95% CI)	Net monetary benefit (95% CI)	No. cost-beneficial cycles
1	97,953	31,494	0.32 (0.29–0.36)	31,494	0.32 (0.29–0.36)	$81.95 ($69.04–$95.45)	$141.05 ($82.92–$199.94)	7
2	64,970	18,951	0.29 (0.25–0.33)	50,445	0.51 (0.47–0.57)	$87.47 ($72.34–$104.28)	$135.53 ($76.31–$196.09)	
3	38,430	11,069	0.29 (0.23–0.34)	61,514	0.63 (0.58–0.69)	$88.01 ($72.01–$108.76)	$134.99 ($70.98–$196.59)	
4	20,216	4,757	0.24 (0.17–0.30)	66,271	0.68 (0.62–0.74)	$106.55 ($82.79–$143.99)	$116.45 ($45.82–$175.79)	
5	10,156	2,616	0.26 (0.16–0.36)	68,887	0.70 (0.65–0.77)	$96.44 ($71.06–$147.05)	$126.56 ($53.09–$187.69)	
6	5,238	1,368	0.26 (0.14–0.41)	70,255	0.72 (0.66–0.78)	$95.39 ($65.14–$171.69)	$127.61 ($36.25–$193.65)	
7	2,601	345	0.13 (0.00–0.30)	70,600	0.72 (0.66–079)	$174.40 ($80.24–$569.19)	$48.60 (–$336.65–$157.49)	
8	1,011	89	0.09 (0.00–0.33)	70,689	0.72 (0.66–0.79)	$237.14 ($63.56–$334.06)	–$14.14 (–$128.66–$184.61)	

Avg., average; CI, confidence interval; Cum., cumulative; LB, live birth; No., number.

Looking at all age groups, as expected, less treatment cycles provided good value for money in older age groups given that cycle-specific live-birth rates were lower. While in women aged <42 years seven or more complete treatment cycles provided good value for money, no cycles provided good value in women aged ≥42 years. Cycles that were identified as cost-beneficial had a NMB ranging from $27 (sixth complete cycle in 40–41-year-olds; 95% CI: –$312.84-$131.60) to $188 per taxpayer (seventh complete cycle in <30-year-olds; 95% CI: $123.78-$244.57). In contrast, for women ≥42 years, treatment costs per taxpayer and live birth were as high as $2,481 whereas each taxpayer only derived an average benefit of $223 per live birth, leading to a net monetary loss of up to $2,258.

### 3.2. Sensitivity analyses

One-way sensitivity analyses show that, generally, variability in the number of fertility treatment cycles conducted annually had the biggest impact on the NMB. This is because the average number of taxpayers funding one fertility treatment cycle, when holding the number of taxpayers constant, changed dramatically (143–1,429 taxpayers/cycle) over the range of values explored. Therefore, compared to the base case, the NMB increased (decreased) with fewer (more) fertility treatment cycles conducted annually, and the impact tended to increase over successive cycles and with increasing age (i.e., as costs per live birth increase).

**Figure 4:**
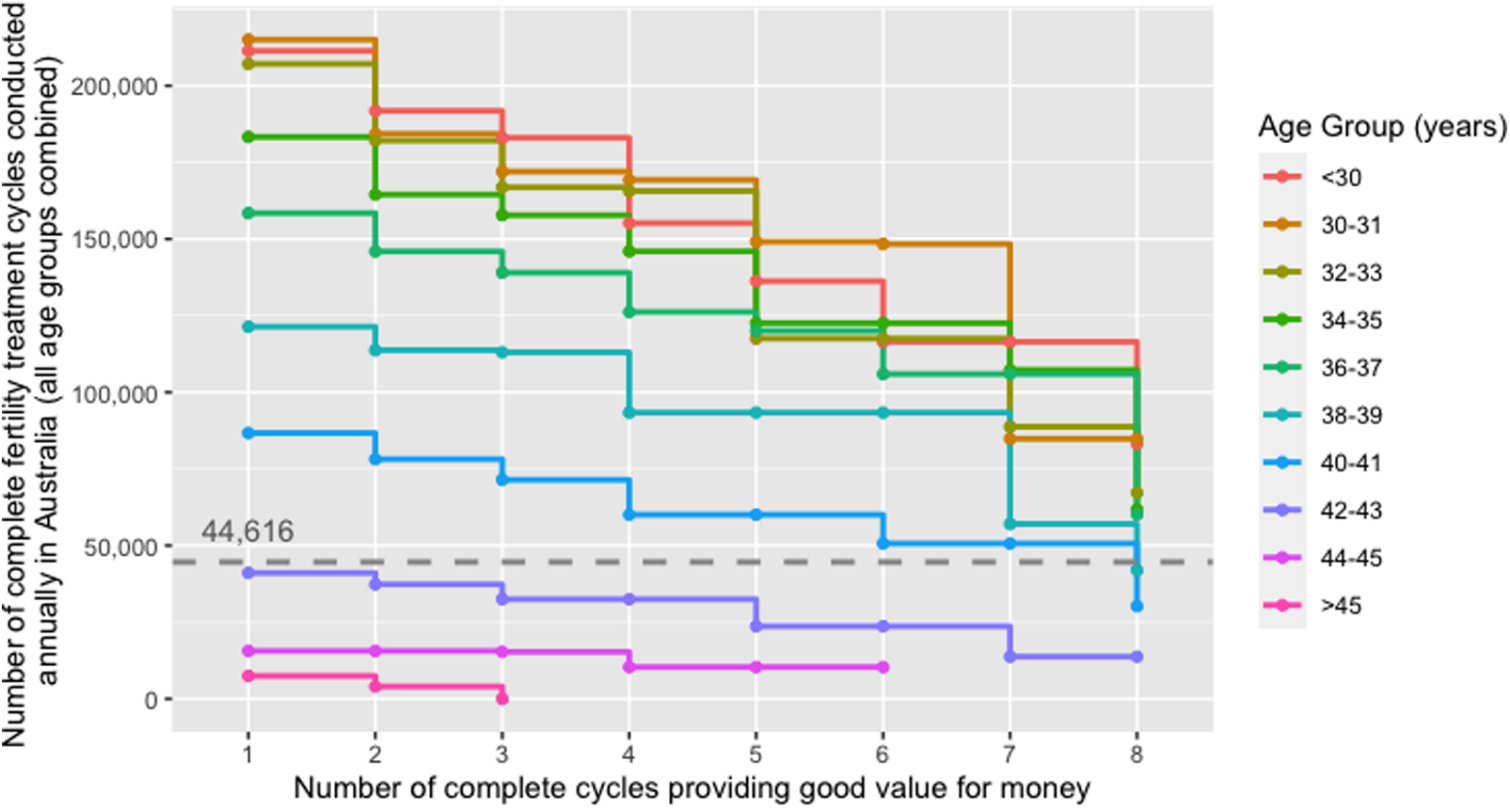
Threshold number of complete fertility treatment cycles conducted annually in Australia beyond which treatment cycles did not provide good value for money from a taxpayer perspective stratified by age group; scenario where only those women who reached the oocyte pick-up procedure were considered. The dashed line indicates the number of complete fertility treatment cycles conducted in the baseline year (i.e., 44,616 complete fertility treatment cycles in 2018). The threshold values for the number of complete fertility treatment cycles conducted annually represent the maximum number of cycles that can be conducted annually in order for the cycle in the respective age group to be cost-beneficial when holding all other parameters constant (e.g., IVF treatment costs, live-birth rates).

Figure 4 shows the threshold values of the number of complete fertility treatment cycles conducted annually in Australia beyond which treatment cycles did not provide good value for money stratified by age group. It reflects the maximum number of cycles conducted annually where IVF cycles were cost-beneficial. In contrast, any number of cycles beyond the threshold value would raise the costs per taxpayer and live birth above the taxpayer VSB, leading to a negative NMB. If <100,000 cycles were conducted annually in Australia, three or more complete treatment cycles provided good value for money in women aged <40 years. For treatment cycles in women ≥42 years to become cost-beneficial, the annual number of complete cycles conducted in Australia had to be <41,073.

Two-way sensitivity analyses indicate that up to six complete cycles were cost-beneficial in younger age groups (<38 years) if more than twice as many fertility treatment cycles were conducted than in the base year 2018 and if, simultaneously, the number of taxpayers was at the lower threshold of 10 million.

**Figure 5:**
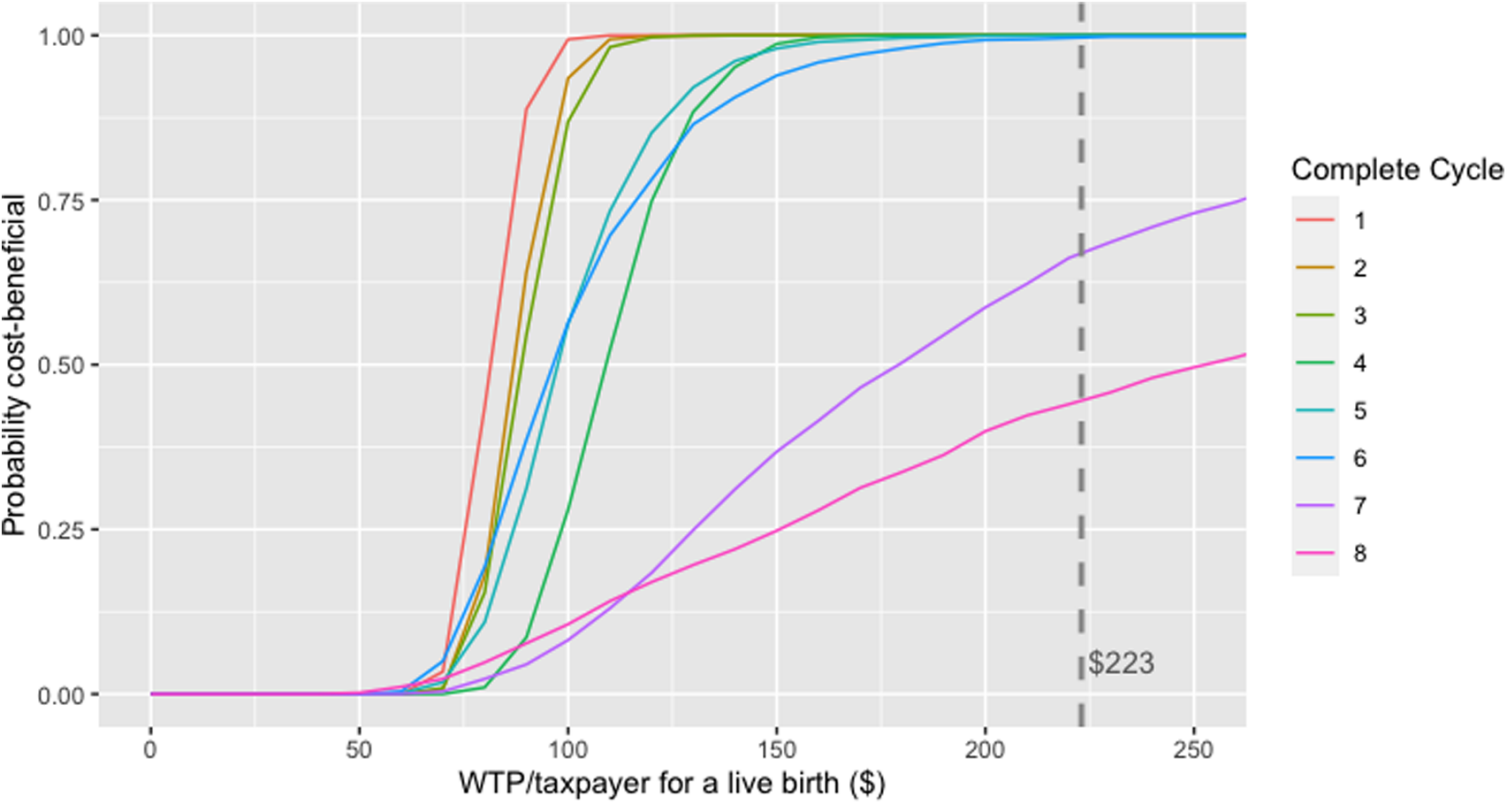
Net-benefit acceptability curve generated from the probabilistic sensitivity analysis in 38–39-year-old women in the scenario where only women reaching oocyte pick-up and their costs were considered.

The PSA suggested a < 50% chance that treatment cycles in women aged ≥42 years provided good value for money at the base-case VSB of $223. In contrast, > 50% and >75% of iterations for women <42 years had a positive NMB for seven or more and five or more complete cycles, respectively, indicating that fertility treatment in these age groups was likely cost-beneficial. Figure 5 provides an example net-benefit acceptability curve for 38–39-year-old women which clearly shows the high probability of six complete IVF cycles being cost-beneficial in this age group at a VSB of $223 per taxpayer.

### 3.3. Incremental analyses

**Figure 6:**
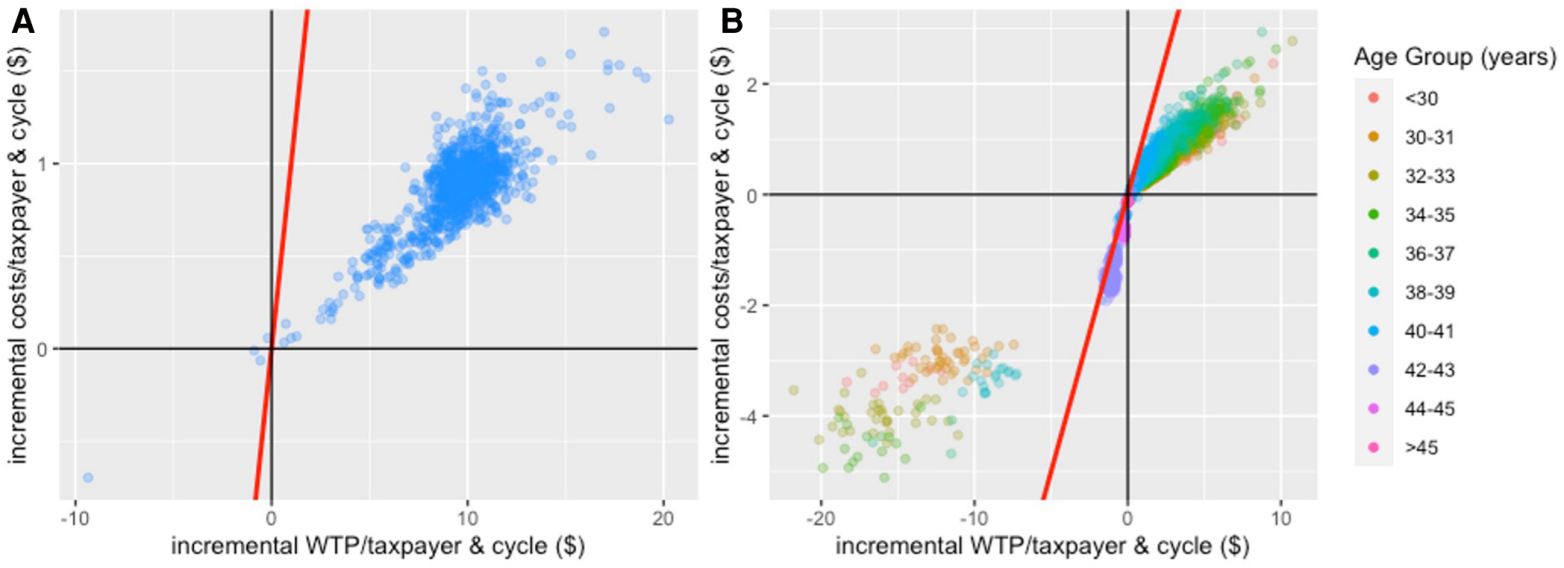
Cost-benefit planes for IVF treatment comparing the intervention package of fertility treatment (i.e., only funding cost-beneficial treatment cycles) to the current package of fertility treatment (comparator) based on the analysis that only considers costs and benefits of women reaching OPU. Panel (**A**): cost-benefit plane for the aggregated analysis; Panel (**B**): cost-benefit plane for the age-stratified analysis. The red line indicates points of indifference (i.e., where incremental costs equal incremental benefits). In Panel (**B**), 1.51% of observations are to the left of the red line in the lower left quadrant: 1.0% of observations in <30-year-old women; 4.4% in 30–31-year-old women; 3.2% in 32–33-year-old women; 1.5% in 34–35-year-old women; 0.3% in 36–37-year-old women; 1.9% in 38–39-year-old women; 0.6% in 40–41-year-old women; 2.2% in 42–43-year-old women; 0% in 44–45-year-old women; 0% in >45-year-old women. These points indicate observations where the current package of fertility treatment would be preferred over the policy intervention package. It is due to a limitation of our approach and Supplementary Appendix C provides a more detailed explanation.

Cost-benefit planes (Figure 6) show a preference for the policy intervention package over the current package of fertility treatment as incremental benefits (approximately $5-$15 per taxpayer) significantly outweighed incremental costs (approximately $0.25-$1.50 per taxpayer) in most model iterations (Supplementary Appendix C explains why the current package was preferred in a small number of model iterations). Generally, for age groups in which most/all cycles were found to be cost-beneficial, there was a positive incremental benefit that came at an additional cost. However, the incremental benefits mostly outweighed additional costs as evidenced by the mass of points below the red line indicating equal incremental costs and benefits. In contrast, in age groups where most/all cycles were found not to be cost-beneficial and, hence, were not funded in the policy intervention package, there were cost savings (negative incremental costs) but taxpayers also missed out on the benefits associated with births in these cycles (negative incremental benefits). Again, most points were located below the red line, meaning cost savings outweighed forgone benefits.

## 4. Discussion

We conducted one of the first CBAs that used DCE results in an economic evaluation using the exemplar of publicly funded IVF treatment in Australia. We found a > 75% likelihood that five or more complete IVF treatment cycles provide good value for money in women aged <42 years. Due to significantly lower live-birth rates in older women (≥42 years) the provision of IVF treatment is unlikely cost-beneficial with average costs per taxpayer and live birth exceeding the base-case VSB of $223 in >50% of model iterations in the PSA. Our incremental analysis, using the status quo of unrestricted partial funding for IVF treatment in Australia as the comparator, suggests that only funding cost-beneficial treatment cycles is preferred from an economic perspective. However, healthcare funding decisions should be based on a broader set of criteria than simply economic arguments with decision-makers also considering and weighting effectiveness, safety, and equity concerns (92, 93). Our evidence provides important information on benefits relative to costs of fertility treatment for women of different age by reporting the number of cost-beneficial treatment cycles from a taxpayer (i.e., general population) perspective. Therefore, our results can help inform the debate about public and third-party funding arrangements for IVF.

Given the dominance of a CEA framework in HTA, we presented results in a similar way to common CEA outputs. This will facilitate understanding and likely increases acceptance of our findings in the field with the aim of the evidence being considered in policy and funding decisions. This is important because a CEA framework and traditional outcome metrics such as QALYs are inadequate to assess fertility treatment. While CEAs can inform the comparative cost-effectiveness of different types of fertility treatment or treatment regimens (25, 94), they are ill-suited to provide evidence on the value for money of such treatments. We addressed this gap in the literature by performing the first detailed CBA of IVF treatment, which provides important evidence on the number of cycles stratified by age that provide good value from a taxpayer perspective. While fertility treatment was the focus of our study, the methods developed are applicable to other areas of healthcare, and especially for analyses from a taxpayer perspective. A recent government report informing National Health Service (NHS) funding in the United Kingdom (48) demonstrated the feasibility of applying the methods developed theoretically by McIntosh (46), including creating cost-benefit planes and net-benefit acceptability curves. We extended this framework in three important ways: First, our approach shows how WTP values in terms of taxpayer contributions, derived from a DCE, can be incorporated into a CBA to provide evidence on the value for money of government spending from a taxpayer perspective. Second, we explored a complex mix of treatment cycles across age groups with different cost and benefit estimates per taxpayer and developed an approach to combine these into a single measure to use in incremental analyses. Third, we developed an interactive online app to visualize our results on an easy-to-use dashboard. This allows someone without programming skills to explore the results and enhances transparency by providing a comprehensive set of figures as well as detailed labels for data points.

The traditional cost-effectiveness analysis using QALYs and a WTP threshold of $50,000 (95, 96) indicates that the number of cycles representing good value decreases continuously with age, from 7 cycles in women aged <30 years to 1 cycle in women aged 38–39 years and no cycles providing good value in women aged ≥40 years. While it is not possible to directly compare the results with the CBA due to methodological differences, this comparison supports the argument that QALYs provide a narrower measure of benefits compared to the CBA/VSB. Furthermore, the cost-effectiveness results are not generally in line with current funding arrangements of IVF in most countries which provide funding for multiple cycles until at least 40 years of age.

### 4.1. Limitations

The use of a taxpayer perspective required that we consider current utilization (i.e., number of fertility treatment cycles performed within baseline year) and number of taxpayers in Australia within our analyses. This limits the generalizability of our results to other countries and contexts who would need to use their own treatment utilization rates and number of taxpayers to assess value for money. However, we explored the impact of both factors extensively in sensitivity analyses: While the NMB and number of cost-beneficial cycles changes significantly depending on the number of taxpayers and cycles performed annually in some age groups, our main conclusions are relatively stable at the extremes of number of cycles and taxpayers.

A second limitation is that fertility treatment can incur additional costs not considered in our analyses such as costs associated with side effects (e.g., ovarian hyperstimulation syndrome) or unsuccessful treatment (e.g., miscarriage, stillbirth). Incorporating these additional costs is expected to reduce the NMB of treatment cycles but is unlikely to significantly change our main conclusions. This is because our NMB estimates for cost-beneficial cycles are >$25 per taxpayer and approximately 80% of clinical pregnancies result in a live birth while severe side effects such as ovarian hyperstimulation syndrome are now extremely rare (58).

Lastly, we were not able to take into account any deadweight loss that inevitably results from taxation of individuals or businesses (97). Our analyses only account for the contributions of an average taxpayer to fund IVF treatment as well as the benefits associated with successful treatment.

## 5. Conclusion

This study uses DCE results in an economic evaluation framework for fertility treatment. The provision of fertility treatment is valued highly by taxpayers and at least five publicly funded treatment cycles seem to provide good value for money in most age groups. The CBA method provides a novel approach to incorporating WTP values in HTA and the results can help inform discussions about treatment funding arrangements.

## Data Availability

The data analyzed in this study is subject to the following licenses/restrictions: The datasets analyzed for this study are not publicly available due to ethical restrictions. Aggregated data is published via an accompanying online app. Requests to access these datasets should be directed to Elena Keller, e.keller@unsw.edu.au.
