# Supplementary material for: Does in vitro fertilization (IVF) treatment provide good value for money? A cost-benefit analysis
Source: Front Glob Womens Health. 2023 Mar 1;4:971553. doi: 10.3389/fgwh.2023.971553 (PMC10014591; doi:10.3389/fgwh.2023.971553)
Supplement: Supplementary file 1 [file Datasheet1.pdf]

# *Supplementary Material*

## Appendix A: Markov model used for cost-benefit analysis

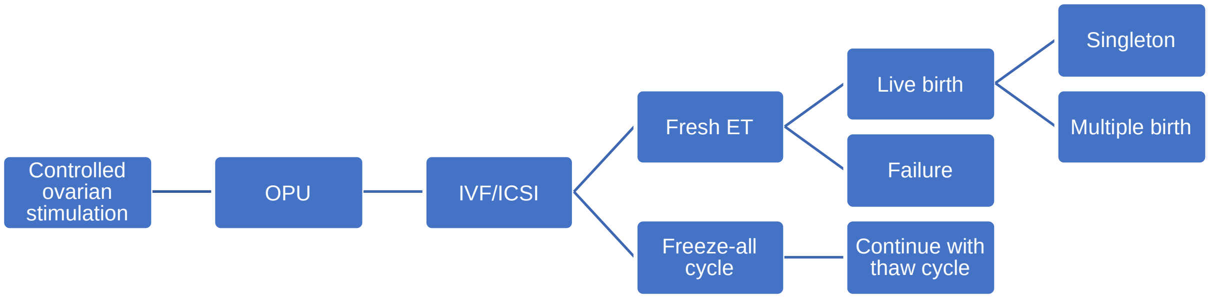


**Supplementary Figure 1.** Illustration of clinically and economically relevant stages of a fresh IVF treatment cycle considered in the Markov model.

Notes: ET, embryo transfer; ICSI, intracytoplasmic sperm injection; IVF, *in vitro* fertilization; OPU, oocyte pick-up. A cycle can fail at all stages (e.g., no oocytes available after OPU) in which case it is possible to continue with a fresh or thaw cycle, and/or women can permanently drop out of treatment (absorbing state).

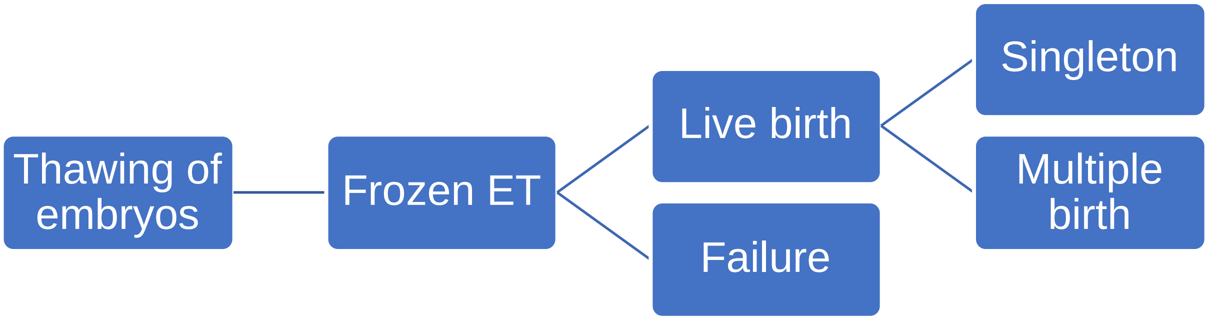


**Supplementary Figure 2.** Illustration of clinically and economically relevant stages of a thaw IVF treatment cycle considered in the Markov model.

Notes: ET, embryo transfer. A cycle can fail at all stages (e.g., no clinical pregnancy after frozen ET) in which case it is possible to continue with a fresh or thaw cycle, and/or women can permanently drop out of treatment (absorbing state).

**Appendix B: Additional details about the discrete choice experiment used to derive the value of a statistical baby (VSB)**

**Design:** We derived willingness-to-pay (WTP) values for a live birth (i.e., VSB estimate) from a discrete choice experiment (DCE) conducted in Australia in which participants had to choose their preferred treatment in 12 choice scenarios. Each choice scenario presented two fertility treatments described by seven attributes (treatment success rate, side effects, counselling/peer support, treatment journey, continuity of care, availability of experimental treatments, and treatment cost) which have been identified using qualitative work (i.e., literature review, focus group discussions, and expert consultations). Supplementary Table 1 provides an overview of the attributes and their levels used in the DCE. An online pilot study was conducted to validate the appropriateness of the seven fertility treatment attributes and their levels. The results of this pilot informed the D-efficient Bayesian fractional experimental design of the final questionnaire for the DCE. The questionnaire was structured as follows: 1.) background information about fertility treatments and their funding in Australia; 2.) one practice choice scenario followed by 12 hypothetical choice scenarios (i.e., the actual DCE); 3.) socio-demographic questions.

**Data collection:** The final DCE was administered online to 801 English-speaking Australian citizens or residents aged 18 years or over through a commercial survey company (Survey Sampling International). The sample was representative of the Australian general population aged 18 years and over in terms of gender, age, and marital status. Supplementary Table 2 compares socio-demographic characteristics of participants in the DCE and the Australian general population aged 18 years and over.

**Analysis:** Preferences for attribute levels were derived based on a regression framework using classical conditional logit, multinomial logit and generalized multinomial logit models. Regression results of the preferred model (generalized multinomial logit model) were used to derive WTP values for fertility treatment attributes and their levels according to the marginal rate of substitution with the cost attribute.

**Justification why only one attribute was used in the cost-benefit analysis (CBA):** Within the CBA framework to assess value for money of IVF treatment, monetization of the benefits of IVF treatment was required which were then compared to the cost of treatment. The objective, and thus benefit, of IVF is the birth of a baby, and thus we used the WTP estimate for a 1% improvement in the live birth rate per cycle, obtained from the DCE study, to derive a VSB estimate (i.e., WTP for a live birth). It would be possible to include the other attributes (e.g., side effects), but HTA funding decisions regarding IVF are based on the chance of having a baby (a proxy of female age and number of cycles is used), and thus the 1% improvement in the live birth rates was appropriate.

**Supplementary Table 1.** Fertility treatment attributes and their levels used in the discrete choice experiment.

| Attribute | Levels |
|---|---|
| **Treatment success rate** | 10% chance of having a baby<br>20% chance of having a baby<br>40% chance of having a baby |
| **Side effects** | Negligible, causing no problems performing usual activities<br>Moderate, causing some problems performing usual activities<br>Significant, causing major problems performing usual activities |
| **Counselling and peer support** | No information on counselling or peer support groups provided<br>Brochures provided on external counselling and peer support groups<br>Onsite clinic counsellors and peer support groups provided |
| **Treatment journey** | Same treatment pathway for all patients<br>Some level of personalized treatment based on upfront tests<br>High level of tailored, personalized treatment based on detailed patient tests |
| **Continuity of care** | Patients are seen by different doctors and nurses at each visit<br>Patients are seen by the same doctor but different nurses at each visit<br>Patients are seen by the same team of fertility doctors and nurses at each visit |
| **Availability of 'alternative' experimental treatments** | No 'alternative' treatments available<br>'Alternative' treatments may be considered if requested by patient<br>'Alternative' treatments offered to all patients |
| **Treatment cost** | No additional annual tax<br>Additional $5 in annual tax<br>Additional $12 in annual tax |

**Supplementary Table 2.** Socio-demographic characteristics of participants in the DCE and the Australian general population aged 18 years and over (based on 2016 Australian Census).

| Category | Sample: n (%) | Australia: (%) |
|---|---|---|
| **Gender** | | |
| Male | 389 (49%) | (49.3%) |
| Female | 411 (51%) | (50.7%) |
| Other | 1 (<1%) | |
| **Age range (years)** | | |
| 18-24 | 84 (11%) | 15-24[a] (12.8%) |
| 25-34 | 162 (20%) | (14.4%) |
| 35-40 | 112 (14%) | 35-39[a] (13.5%) |
| 41-54 | 233 (29%) | 40-54[a] (20.1%) |
| 55-64 | 131 (16%) | (11.8%) |
| ≥65 | 79 (10%) | (15.8%) |
| **Marital status** | | |
| Never married | 251 (31%) | |
| Married | 367 (46%) | (47.7%) |
| De facto | 69 (9%) | |
| Separated/divorced | 82 (10%) | (11.7%) |
| Widowed | 24 (3%) | (5.2%) |
| Prefer not to say | 8 (1%) | |

Notes: [a]The 2016 Australian Census data (http://quickstats.censusdata.abs.gov.au/census_services/getproduct/census/2016/quickstat/036) use slightly different age ranges. The closest range is provided in the table.

## Appendix C: Incremental analysis – Methods

**Comparator:** We conducted an incremental cost-benefit analysis (CBA) using the current funding environment in Australia as the comparator. This allowed us to construct incremental cost-benefit ratios (ICBR) following the approach outlined in McIntosh (1). We defined the comparator as funding the current "package" of fertility treatment in Australia, which is characterized by providing funding for an unlimited number of cycles in all age groups. To facilitate the analysis, we derived the costs and benefits per taxpayer of an average cycle in this package according to Equation 1 below: Using utilization data for 2018 (based on the Australia and New Zealand Assisted Reproduction Database (ANZARD)) to determine the proportion of cycles stratified by age group and complete cycle, we calculated the sum of proportionate costs and benefits in each cycle and age group.

**Equation 1:** Calculation of the costs per taxpayer for an average cycle in the current package of fertility treatment.

$$Costs\ per\ taxpayer\ of\ average\ cycle\ in\ current\ package = \sum_{Age\ Group=<30}^{Age\ Group=>45} \sum_{Complete\ Cycle=1}^{Complete\ Cycle=8} \frac{No.cycles_{Age\ Group,Complete\ Cycle}}{\sum\sum No.cycles_{Age\ Group,Complete\ Cycle}} * Costs_{Age\ Group,Complete\ Cycle}$$

Supplementary Table 1 provides an example calculation for the proportionate costs in each cycle and age group for the current package of fertility treatment. The column "Proportion of all women" was populated based on utilization data for 2018 from ANZARD. Due to the lack of appropriate data on treatment costs for IVF cycles performed in 2018, we assigned average costs per complete cycle and taxpayer based on the model outputs from the probabilistic sensitivity analyses. In the example, we used cost figures resulting from one iteration of the Markov model using Monte Carlo simulation for 1,000 women in each age group. Here, a first complete cycle in <30-year-old women costs $35.26 per taxpayer and 2.161% of all cycles conducted annually in Australia represent first complete cycles in this age group. Therefore, we considered $0.76 (2.161% x $35.26) in the calculation for the costs per taxpayer of an average cycle in the current package; i.e. $0.76 is the first summand in the calculation of the costs for an average cycle. The other summands are calculated equivalently and represent the costs in all other cycles and age groups (see column "Proportionate costs" in Supplementary Table 1).

**Supplementary Table 1.** Example calculation for the average costs each taxpayer is funding in the current package of fertility treatment.

| Age group (years) | Complete cycle | Proportion of all women | Avg. costs per taxpayer and cycle (example from probabilistic sensitivity analysis) | Proportionate costs |
|---|---|---|---|---|
| **<30** | 1 | 2.161% | $35.26 | $0.76<br>(i.e., 2.161% x $35.26) |
| **<30** | 2 | 5.238% | $32.21 | $1.69 |
| **<30** | 3 | 2.062% | $32.93 | $0.68 |
| **<30** | 4 | 0.767% | $29.91 | $0.23 |
| **<30** | 5 | 0.297% | $28.56 | $0.08 |
| **<30** | 6 | 0.109% | $28.30 | $0.03 |
| **<30** | 7 | 0.056% | $33.11 | $0.02 |
| **<30** | 8 | 0.034% | NA (0 women starting cycle) | $0.01<br>(i.e., 0.034% x $33.11) |
| **30-31** | 1 | 1.905% | $34.43 | $0.66 |
| **30-31** | 2 | 4.608% | $31.74 | $1.46 |
| **…** | … | … | … | |
| **44-45** | 7 | 0.037% | $21.47 | $0.01 |
| **44-45** | 8 | 0.049% | NA (excluded from analysis due to lack of sufficient data) | $0.01<br>(i.e., 0.049% x $21.47) |
| **>45** | 1 | 0.121% | $20.74 | $0.03 |
| **>45** | 2 | 0.293% | $20.63 | $0.06 |
| **>45** | 3 | 0.121% | $20.34 | $0.02 |
| **>45** | 4 | 0.064% | NA (excluded from analysis due to lack of sufficient data) | $0.01<br>(i.e., 0.064% x $20.34) |
| **>45** | 5 | 0.033% | NA (excluded from analysis due to lack of sufficient data) | $0.01<br>(i.e., 0.033% x $20.34) |
| **>45** | 6 | 0.010% | NA (excluded from analysis due to lack of sufficient data) | $0.00<br>(i.e., 0.010% x $20.34) |
| **>45** | 7 | 0.008% | NA (excluded from analysis due to lack of sufficient data) | $0.00<br>(i.e., 0.008% x $20.34) |
| **>45** | 8 | 0.002% | NA (excluded from analysis due to lack of sufficient data) | $0.00<br>(i.e., 0.002% x $20.34) |
| **Total** | | **100%** | | **$28.42** |

In some cases, it was not possible to derive the average costs per taxpayer and treatment cycle from the probabilistic sensitivity analysis output which was due to one of two reasons:

1) The treatment cycle was excluded from the analysis due to a lack of sufficient data on transition probabilities in ANZARD (e.g., 8$^{th}$ complete cycle in 44-45-year-old women was excluded because available data was based on <30 women).

2) No women started the treatment cycle in the iteration of the Markov model considered due to the model terminating (e.g., if women achieved 4 live births) or due to women dropping out of treatment prior to reaching the treatment cycle considered.

In these cases, we assumed that average costs per taxpayer and cycle were equal to the average costs per taxpayer and cycle from the last complete cycle with an available estimate in the age group considered. For instance, in the example shown in Supplementary Table 1, there was no available cost estimate for the 8$^{th}$ complete cycle in <30-year-old women. Therefore, we assumed average costs were the same as in the 7$^{th}$ complete cycle, the last complete cycle with a cost estimate (i.e., $33.11 per taxpayer).

Finally, we summed the proportionate costs (last column in Supplementary Table 1) across all cycles and age groups to obtain the average costs that each taxpayer is funding, on average, for the provision of the current package of fertility treatment. In the example calculation in the table, each taxpayer would contribute a total of $28.42, on average, to fund the current package of fertility treatment.

The calculation for the benefits of an average cycle in the current package is equivalent: Average benefits per taxpayer and complete cycle are based on the model outputs from the probabilistic sensitivity analysis and calculated as the cycle-specific live birth rate multiplied by the willingness-to-pay for a live-born baby – the value of a statistical baby. Where such an estimate was not available for a treatment cycle, it was assumed that average benefits per taxpayer are the same as in the last cycle with a benefit estimate. Proportionate benefits are then calculated as the average benefits per taxpayer and cycle multiplied by the proportion of all women in that age group and cycle. Finally, the average benefits per taxpayer for the current package of fertility treatment are derived by summing the proportionate benefits across all cycles and age groups. For the example shown in the table, the average benefits per taxpayer for the current package of fertility treatment were estimated to be $92.08.

**Policy intervention:** The 'policy intervention' which we compare to the current funding arrangement in Australia is defined in this study as funding the number of cycles that were deemed cost-beneficial in the CBA (i.e., those that have a positive NMB) by female age. To determine the sum of proportionate costs and benefits in each cycle and age group in this policy intervention package we used the same utilization data (for 2018; based on ANZARD). However, prior to determining the proportion of cycles stratified by age group and complete cycle, we excluded any complete cycles that were not found to provide good value for money. Otherwise, calculations were the same. To continue the example above (Supplementary Table 1), the cost and benefit estimates for the provision of the policy intervention package of fertility treatment in the particular iteration of the probabilistic sensitivity analysis considered were as follows:

- The average costs that each taxpayer is funding were $29.22.
- The average benefits that each taxpayer is receiving were $101.33.

**Incremental cost-benefit analysis:** In the final step, we determined the incremental cost and the incremental benefit as the difference in average costs and benefits per taxpayer, respectively, between

the policy intervention and the current package of fertility treatment (Supplementary Table 2). Wherever, incremental benefits outweigh incremental costs, we would prefer the policy intervention package of fertility treatment. Similarly, we would prefer the current package of fertility treatment where this is not the case. For the example above, incremental costs are $0.80 (i.e., $29.22 - $28.42) and incremental benefits are $9.25 (i.e., $101.33 - $92.08), meaning incremental benefits outweigh incremental costs and, therefore, the policy intervention package of fertility treatment is preferred over the current package of fertility treatment.

Similar to all other analyses (in the main manuscript), we conducted the incremental analyses for two scenarios:

1) We excluded women and their associated costs in each complete cycle who did not reach oocyte pick-up.

2) We considered all women simulated in the model (i.e., 1,000 women per age group in each of the 1,000 iterations of the Markov model in the probabilistic sensitivity analyses) and their associated costs.

In addition to presenting the results of the incremental analysis aggregated for all age groups, we created cost-benefit planes showing the incremental costs and benefits separately for each age group. The respective calculations are equivalent to the aggregated analysis. Supplementary Table 2 includes the (incremental) cost and benefit estimates of the age-stratified analysis corresponding to the example discussed above. The aim of this approach is to show how each age group contributes to the aggregated results, which is why the cost and benefit estimates stratified by age always sum to the aggregated estimates.

For instance, in the example shown in Supplementary Table 2 it can be seen that for women aged <42 years the policy intervention package of fertility treatment provides incremental benefits at additional costs compared to the current package, and that the incremental benefits outweigh the incremental costs. In contrast, for women aged 42-43 years, cost savings in the policy intervention package of fertility treatment are associated with a reduction in benefits. While at least one complete cycle was good value for money and, hence, considered in the policy intervention package, some cycles were not good value and, therefore, excluded. As a result, incremental costs and benefits compared to the current package are negative because the cycles that were excluded were associated with a particular treatment cost and chance of a live birth (i.e., benefit). In Supplementary Table 2 it can also be seen that no cycles in women aged 44-45 years or >45 years in this example were good value, leading to cost and benefit estimates equal to $0 for the policy intervention package. In such cases, incremental costs and benefits would always be negative for the same reason as described above.

**Supplementary Table 2.** Incremental costs and benefits of the policy intervention package of fertility treatment (intervention) compared to the current package of fertility treatment (comparator).

| | Cost estimate | Benefit estimate | Preferred package of fertility treatment |
|---|---|---|---|
| **Aggregated analysis** | | | |
| Current package of fertility treatment (Comparator) | $28.42 | $92.08 | |
| Policy intervention package of fertility treatment (Intervention) | $29.22 | $101.33 | |
| **Incremental analysis** | **$0.80** | **$9.25** | **Intervention package** |
| **Age-stratified analysis** | | | |
| <30 years; comparator | $3.50 | $15.45 | |
| <30 years; policy intervention | $3.90 | $17.17 | |
| **Incremental analysis** | **$0.40** | **$1.71** | |
| 30-31 years; comparator | $3.04 | $12.97 | |
| 30-31 years; policy intervention | $3.37 | $14.46 | |
| **Incremental analysis** | **$0.33** | **$1.49** | |
| 32-33 years; comparator | $4.13 | $17.16 | |
| 32-33 years; policy intervention | $4.61 | $19.15 | |
| **Incremental analysis** | **$0.48** | **$2.00** | |
| 34-35 years; comparator | $4.80 | $18.16 | |
| 34-35 years; policy intervention | $5.33 | $20.27 | |
| **Incremental analysis** | **$0.53** | **$2.11** | |
| 36-37 years; comparator | $4.30 | $13.62 | |
| 36-37 years; policy intervention | $4.80 | $15.20 | |
| **Incremental analysis** | **$0.50** | **$1.58** | |
| 38-39 years; comparator | $3.59 | $8.69 | |
| 38-39 years; policy intervention | $3.95 | $9.70 | |
| **Incremental analysis** | **$0.36** | **$1.01** | |
| 40-41 years; comparator | $2.67 | $4.50 | |
| 40-41 years; policy intervention | $2.92 | $5.02 | |
| **Incremental analysis** | **$0.25** | **$0.52** | |
| 42-43 years; comparator | $1.57 | $1.22 | |
| 42-43 years; policy intervention | $0.34 | $0.36 | |
| **Incremental analysis** | **–$1.24** | **–$0.86** | |
| 44-45 years; comparator | $0.69 | $0.30 | |
| 44-45 years; policy intervention | $0 | $0 | |
| **Incremental analysis** | **–$0.69** | **–$0.30** | |
| >45 years; comparator | $0.13 | $0.02 | |
| >45 years; policy intervention | $0 | $0 | |
| **Incremental analysis** | **–$0.13** | **–$0.02** | |
| **Incremental analysis** | **$0.80** | **$9.25** | **Intervention package** |

Notes: Due to rounding error incremental cost and benefit values might differ slightly from the difference of tabulated values of the cost and benefit estimate.

**Cost-benefit plane:** To visualize the results of the incremental analyses, we constructed cost-benefit planes, which plot the incremental benefits on the horizontal axis and incremental costs on the vertical axis. For both scenarios ((1) only women reaching oocyte pick-up and their costs are considered; (2) all women and their costs are considered) we created two cost-benefit planes:

1) A cost-benefit plane for the aggregated analysis where each of the 1,000 iterations of the model in probabilistic sensitivity analyses is represented by one dot (Panel A of Supplementary Figure 1).

2) A cost-benefit plane for the age-stratified analysis where each of the 1,000 iterations of the model in probabilistic sensitivity analyses is represented by 10 dots – one for each age group (Panel B of Supplementary Figure 1).

As a general rule, interventions are considered to be preferred to the comparator if incremental benefits outweigh incremental costs, and we would be indifferent between the intervention and comparator if incremental costs equal incremental benefits. We visualize the points of indifference in the cost-benefit plane by a straight red line through the origin with slope=1. All dots to the right of this red line indicate that the intervention would be preferred to the comparator as incremental benefits are greater than incremental costs.

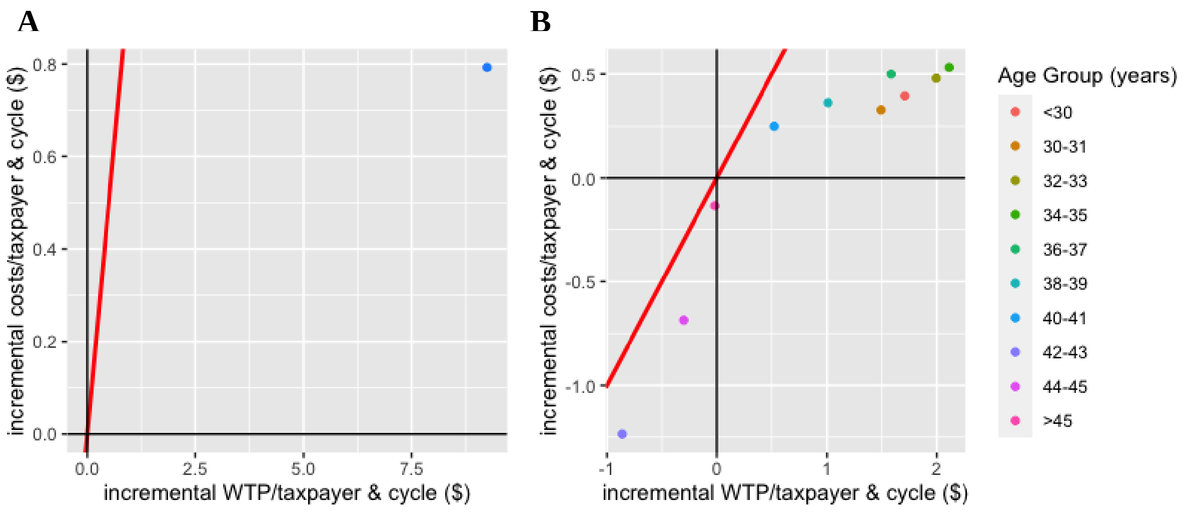


**Supplementary Figure 1.** Cost-benefit planes for the example discussed in the appendix.

Panel A: cost-benefit plane for the aggregated analysis; Panel B: cost-benefit plane for the age-stratified analysis. The red line indicates points of indifference (i.e., where incremental costs equal incremental benefits).

**Limitations:** It was not always possible to assign average costs and benefits per taxpayer for a treatment cycle in the current package of fertility treatment (i.e., comparator) based on the probabilistic sensitivity analysis. This was due to one of two reasons: (1) The treatment cycle was

excluded from the analysis due to a lack of sufficient data on transition probabilities in ANZARD (e.g., 8th complete cycle in 44-45-year-old women was excluded because available data was based on <30 women). (2) No women started the treatment cycle in the iteration of the Markov model considered due to the model terminating (e.g., if women achieved 4 live births) or due to women dropping out of treatment prior to reaching the treatment cycle considered. In such cases, we assumed the cost and benefit estimates were equal to the estimates of the last cycle in this age group with such estimates available. Consequently, if that cycle was considered good value for money, the cycle with missing estimates would also be considered good value. However, given that it is not included in the policy intervention package, the cost-benefit plane might show a preference of the current package of fertility treatment in these age groups/in the aggregated analysis over the policy intervention package.

## References


1. McIntosh E. Using Discrete Choice Experiments within a Cost-Benefit Analysis Framework: Some Considerations. *PharmacoEconomics* (2006) 24(9):855-68. doi: https://doi.org/10.2165/00019053-200624090-00004.

## Appendix D: Cost-effectiveness analysis of IVF treatment

## 1 Methods

We conducted an additional cost-effectiveness analysis of IVF treatment using the same Markov model structure as for the cost-benefit analysis and simulating n=10,000 women. As outcome measures, we reported both average costs per live birth and average costs per maternal quality-adjusted life-year (QALY) gained. Model input parameters remained unchanged (including costs and transition probabilities) except for the treatment benefit measure: For the cost-benefit analysis, benefits of IVF treatment (i.e., a live birth) were valued using the value for a statistical baby, whereas in the cost-effectiveness framework, we measured benefits using maternal QALYs. To obtain incremental cost-effectiveness ratios (ICER), we compared publicly funded IVF treatment (intervention) to doing nothing (comparator) and assume that all women in the comparator group are infertile and remain involuntarily childless.

Consistent with one of only few studies reporting cost per QALY estimates for fertility treatment (1), we used a 20-year time horizon over which benefits are accrued and discounted future QALYs at a rate of 3.5% per year. Costs were not discounted because we aimed to find the level of public funding for IVF treatment that is in line with taxpayer preferences which is independent of the time horizon.

We made the following assumptions:

1. All women survive the 20-year time horizon.

2. All women had no children prior to starting IVF treatment.

3. All women want to have four children.

4. There is no QALY loss after a miscarriage or stillbirth.

More specifically, we used the QALY utility weights summarized in Supplementary Table 1, which are partly consistent with the analysis by Scotland et al. (1). Unlike Scotland et al. (1), however, our analysis required varying maternal utility weights depending on the number of live births achieved with the help of IVF treatment. These more detailed utility weights were obtained from the literature (2).

**Supplementary Table 1.** QALY utility weights used to parameterize Markov model.

| | Base-case value | Source |
|---|---|---|
| **Being infertile with no children** | 0.792 | (2) |
| **Being infertile with 1 child** | 0.845 | (2) |
| **Being infertile with 2 children** | 0.8565 | Imputed as mid-point between being infertile having 1 and 3 children |
| **Being infertile with 3 children** | 0.868 | (2) |
| **Population norm (i.e., being fertile/having 4 children)** | 0.93 | (1) |

The ICER for publicly funded IVF treatment stratified by complete cycle and female age was derived according to the following equation:

$$ICER = \frac{Government\ cost_{Intervention} - Government\ cost_{Comparator}}{Total\ QALYs_{Intervention} - Total\ QALYs_{Comparator}}$$

We only performed a base-case analysis for the main scenario (i.e., where only women who reached oocyte pick-up (OPU) procedure in the Markov model and their associated costs were considered in each complete cycle) for comparison with our main analysis – the cost-benefit analysis.

While our main goal in performing the cost-effectiveness analysis was to ensure comparability with the CBA results, some modifications in the methodology were necessary that impacted comparisons: (1) We assumed a 20-year time horizon over which QALY improvements following a live birth resulting from IVF treatment were considered and discounted future QALYs at a rate of 3.5% per year. In contrast, discounting was inconsequential for the CBA and benefits resulting from each live birth were realized immediately (valued monetarily using the VSB), rather than accumulated over time. (2) In the CBA, each live birth resulted in the same benefit (equal to the VSB of $223), whereas the literature suggests that QALY utility weights do not increase linearly with the number of children (2). Given limited evidence, we had to make strong assumptions about these utility weights and the preferences of women, which were not required for the CBA.

## 2 Results

Supplementary Table 2 reports costs per live birth achieved and costs per QALY gained stratified by female age group and complete cycle and Supplementary Figure 1 provides a visual representation of ICER values against the generally assumed QALY threshold value in Australia of approximately $50,000 (3, 4). The results indicate that the number of cycles with good value for money (i.e., with a cost per QALY gained estimate below the $50,000 threshold value) continuously declines with female age with seven cycles providing good value for money in women aged <30 years compared to one cycle in women aged 38-39 years. In women aged 40 years and over no cycles were found to provide good value for money.

**Supplementary Table 2.** Costs per live birth (LB) and costs per quality-adjusted life-year (QALY) gained for publicly funded IVF treatment stratified by female age group and complete cycle.

| Age group (years) | Complete cycle | Cost per LB | Cost per QALY gained | No. cycles with good value for money* |
|---|---|---|---|---|
| **<30** | **1** | $14,958.37 | $23,917.72 | 7 |
| | **2** | $16,351.55 | $38,036.99 | |
| | **3** | $17,412.51 | $42,007.17 | |
| | **4** | $20,814.33 | $47,936.38 | |
| | **5** | $23,787.00 | $46,316.84 | |
| | **6** | $24,678.68 | $46,853.03 | |
| | **7** | $9,777.07 | $19,081.20 | |
| | **8** | $40,557.04 | $97,310.72 | |
| **30-31** | **1** | $14,726.47 | $23,937.25 | 6 |
| | **2** | $16,929.10 | $39,047.18 | |
| | **3** | $18,485.90 | $44,265.84 | |
| | **4** | $18,781.67 | $41,827.80 | |
| | **5** | $22,051.04 | $44,461.83 | |
| | **6** | $21,533.06 | $45,978.07 | |
| | **7** | $38,510.10 | $71,237.62 | |
| | **8** | $65,177.16 | $71,465.41 | |
| **32-33** | **1** | $15,360.24 | $24,861.41 | 4 |
| | **2** | $17,278.92 | $38,877.20 | |
| | **3** | $18,926.24 | $44,380.30 | |
| | **4** | $19,491.57 | $46,327.67 | |
| | **5** | $25,676.09 | $56,934.10 | |
| | **6** | $27,144.31 | $60,560.60 | |
| | **7** | $29,659.09 | $64,078.89 | |
| | **8** | $30,685.01 | $96,574.98 | |
| **34-35** | **1** | $17,236.78 | $26,839.56 | 3 |
| | **2** | $19,336.92 | $40,922.17 | |
| | **3** | $19,970.69 | $46,718.40 | |
| | **4** | $22,099.42 | $55,692.01 | |
| | **5** | $25,169.40 | $61,534.34 | |
| | **6** | $23,947.62 | $53,738.88 | |
| | **7** | $36,324.69 | $73,883.53 | |
| | **8** | $40,198.17 | $110,893.83 | |
| **36-37** | **1** | $19,998.90 | $30,099.44 | 2 |
| | **2** | $21,295.43 | $42,186.27 | |
| | **3** | $23,030.77 | $53,135.55 | |
| | **4** | $24,670.82 | $59,333.74 | |
| | **5** | $27,457.78 | $65,713.02 | |
| | **6** | $32,623.72 | $72,742.61 | |
| | **7** | $39,705.73 | $92,085.75 | |

| Age group (years) | Complete cycle | Cost per LB | Cost per QALY gained | No. cycles with good value for money* |
|---|---|---|---|---|
| | **8** | $53,813.47 | $104,673.31 | |
| **38-39** | **1** | $26,098.55 | $37,859.49 | 1 |
| | **2** | $28,740.93 | $52,370.09 | |
| | **3** | $28,013.71 | $56,457.69 | |
| | **4** | $34,389.52 | $78,151.13 | |
| | **5** | $32,125.10 | $71,523.88 | |
| | **6** | $31,176.92 | $64,921.66 | |
| | **7** | $75,587.81 | $153,475.57 | |
| | **8** | $103,837.07 | $286,453.08 | |
| **40-41** | **1** | $35,912.19 | $50,127.27 | 0 |
| | **2** | $41,845.49 | $66,714.31 | |
| | **3** | $43,969.16 | $80,874.04 | |
| | **4** | $56,300.13 | $103,405.63 | |
| | **5** | $40,216.68 | $82,637.82 | |
| | **6** | $69,548.30 | $151,257.37 | |
| | **7** | $44,992.16 | $111,263.09 | |
| | **8** | $93,729.67 | $219,720.81 | |
| **42-43** | **1** | $76,759.19 | $102,551.07 | 0 |
| | **2** | $88,945.60 | $123,683.59 | |
| | **3** | $97,006.63 | $141,647.15 | |
| | **4** | $97,451.22 | $139,845.85 | |
| | **5** | $132,669.64 | $219,918.70 | |
| | **6** | $89,631.80 | $142,951.87 | |
| | **7** | $221,128.92 | $326,207.39 | |
| | **8** | $211,089.85 | $366,389.37 | |
| **44-45** | **1** | $193,814.81 | $251,760.38 | 0 |
| | **2** | $190,222.90 | $252,820.33 | |
| | **3** | $182,366.28 | $254,631.39 | |
| | **4** | $373,135.21 | $499,182.88 | |
| | **5** | $191,008.27 | $294,398.35 | |
| | **6** | $85,435.05 | $141,167.19 | |
| | **7** | $291,056.97 | $613,536.50 | |
| **>45** | **1** | $436,693.67 | $563,529.84 | 0 |
| | **2** | $614,434.40 | $788,118.97 | |
| | **3** | Inf | Inf | |

Notes: *The number of cycles with good value for money was determined as those cycles with a cost per QALY gained below a QALY threshold value of $50,000. Cost per live birth (LB) and cost per quality-adjusted life-year (QALY) gained is infinite (Inf) in the third complete cycle in women aged >45 years because no live births were recorded in this cycle and age group, meaning that the cost estimate was divided by zero.

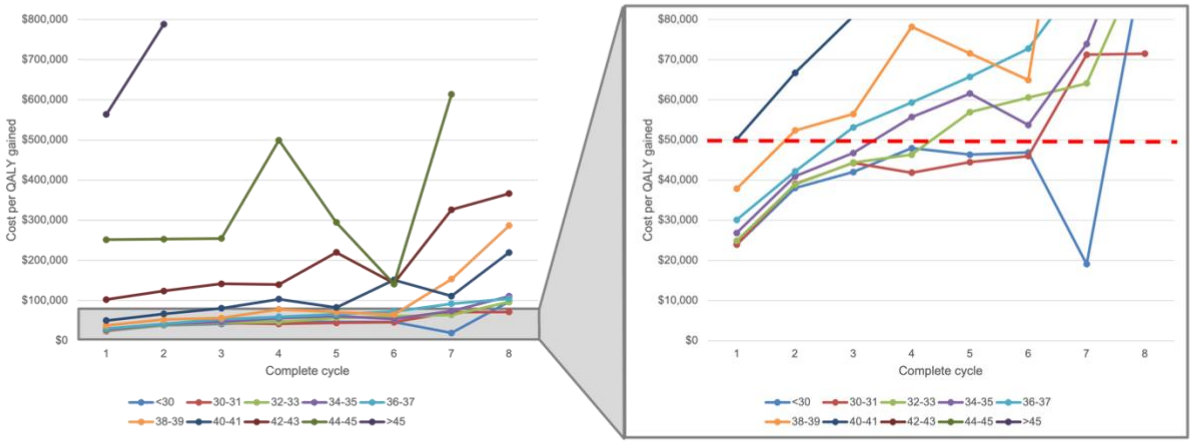


**Supplementary Figure 1.** Government cost per quality-adjusted life-year (QALY) gained by female age group and complete cycle comparing publicly funded IVF treatment to doing nothing resulting in involuntary childlessness.

Notes: The left part of the figure shows cost per QALY estimates for all age groups analyzed; the right part of the figure zooms in on cost per QALY estimates below $80,000. The red dashed line represents the commonly assumed QALY threshold value in Australia of $50,000.

## References

1. Scotland G, McLernon D, Kurinczuk J, McNamee P, Harrild K, Lyall H, et al. Minimising Twins in in Vitro Fertilisation: A Modelling Study Assessing the Costs, Consequences and Cost–Utility of Elective Single Versus Double Embryo Transfer over a 20-Year Time Horizon. *BJOG: An International Journal of Obstetrics & Gynaecology* (2011) 118(9):1073-83. doi: https://doi.org/10.1111/j.1471-0528.2011.02966.x.

2. Krol M, Nap A, Michels R, Veraart C, Goossens L. Health State Utilities for Infertility and Subfertility. *Reproductive Health* (2019) 16(1):47. doi: 10.1186/s12978-019-0706-9.

3. Wang S, Gum D, Merlin T. Comparing the Icers in Medicine Reimbursement Submissions to Nice and Pbac—Does the Presence of an Explicit Threshold Affect the Icer Proposed? *Value in Health* (2018) 21(8):938-43. doi: https://doi.org/10.1016/j.jval.2018.01.017.

4. Lowe A, Dyson S. New Therapies for Advanced Cancers: Can Our Society Afford Them? Is It Ethical to Deny Patients Access to Them? *Actuaries Institute 2013 Actuaries Summit*; Sydney. Sydney: Institute of Actuaries of Australia (2013).

## Appendix E: Tables and figures included in the interactive online app for exemplary age group 38-39 years

**Supplementary Table 1.** Summary statistics for the base-case analysis of the cost-benefit analysis in 38-39-year-old women in the scenario where only women reaching oocyte pick-up and their costs are considered.

Base-case analysis by age group and complete cycle

| | | | Avg. costs/taxpayer for a LB (AU$) | | Net monetary benefit (AU$) | | |
|---|---|---|---|---|---|---|---|
| Age group | Complete cycle | No. women starting cycle | Point estimate | 95% Confidence interval | Point estimate | 95% Confidence interval | No. cycles with good value for money |
| 38-39 | 1 | 97953 | 81.95 | (69.04, 95.45) | 141.05 | (82.92, 199.94) | 7 |
| 38-39 | 2 | 64970 | 87.47 | (72.34, 104.28) | 135.53 | (76.31, 196.09) | 7 |
| 38-39 | 3 | 38430 | 88.01 | (72.01, 108.76) | 134.99 | (70.98, 196.59) | 7 |
| 38-39 | 4 | 20216 | 106.55 | (82.79, 143.99) | 116.45 | (45.82, 175.79) | 7 |
| 38-39 | 5 | 10156 | 96.44 | (71.06, 147.05) | 126.56 | (53.09, 187.69) | 7 |
| 38-39 | 6 | 5238 | 95.39 | (65.14, 171.69) | 127.61 | (36.25, 193.65) | 7 |
| 38-39 | 7 | 2601 | 174.40 | (80.24, 569.19) | 48.60 | (-336.65, 157.49) | 7 |
| 38-39 | 8 | 1011 | 237.14 | (63.56, 334.06) | -14.14 | (-128.66, 184.61) | 7 |

95% confidence intervals were determined using a percentile approach. Avg. indicates average; cum., cumulative; LB, live birth; no., number.

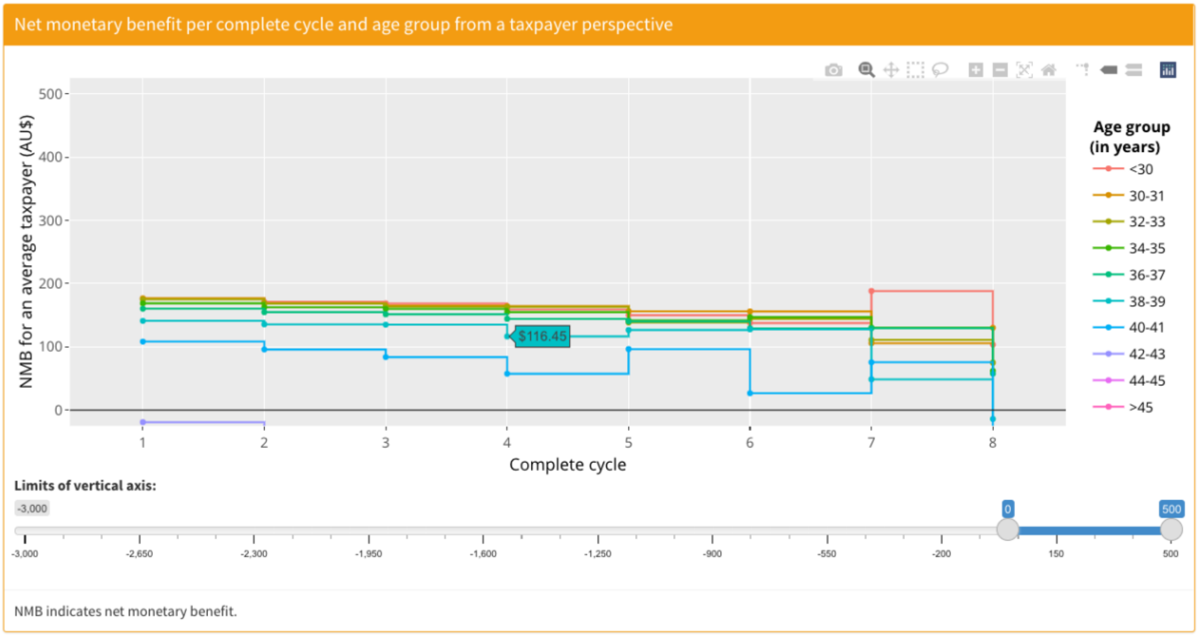


**Supplementary Figure 1.** Net monetary benefit (NMB) per complete IVF treatment cycle and age group from a taxpayer perspective in the base-case analysis.

Notes: The slider at the bottom allows adjusting the limits of the vertical axis to zoom in/out of the figure. When hovering the cursor above data points in the online figure, data labels appear that provide the exact value for the NMB. As an example, the screenshot shows the data label for the 4$^{th}$ complete cycle in 38-39-year-old women ($116.45).

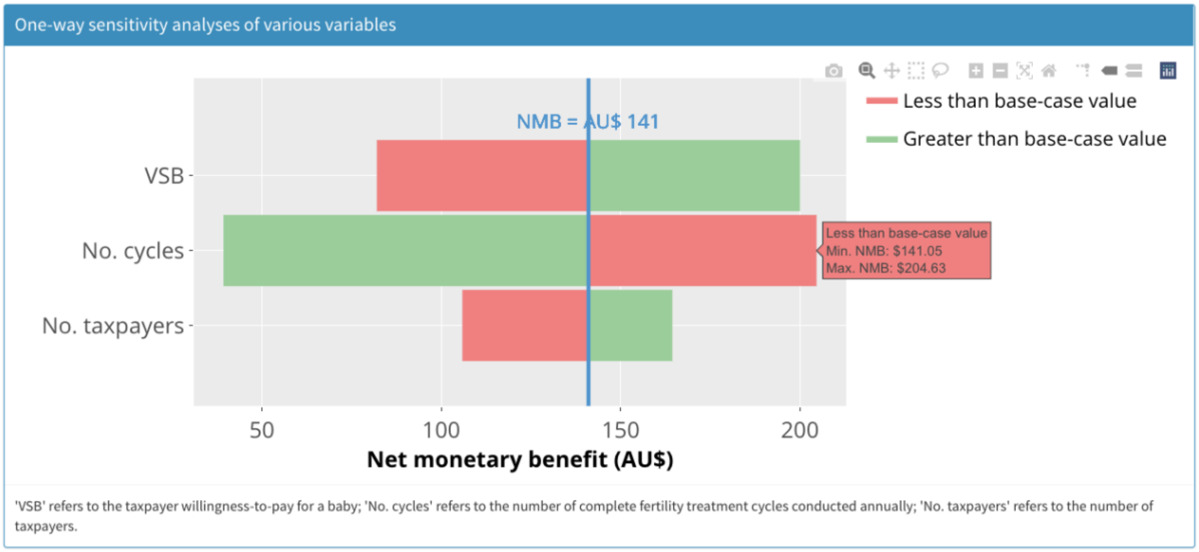


**Supplementary Figure 2.** Tornado diagram for the one-way sensitivity analyses exploring the impact of changes in the (1) value of a statistical baby, (2) number of fertility treatment cycles conducted annually, and (3) number of taxpayers in Australia on the net monetary benefit (NMB) in the 1st complete IVF treatment cycle in 38-39-year-old women.

Notes: When hovering the cursor above the bars in the online figure, data labels appear that indicate the minimum and maximum NMB observed for the values less/greater than the base-case value explored in the respective variable. As an example, the screenshot shows the data label for the case where the number of treatment cycles conducted annually in Australia is less than the base-case value.

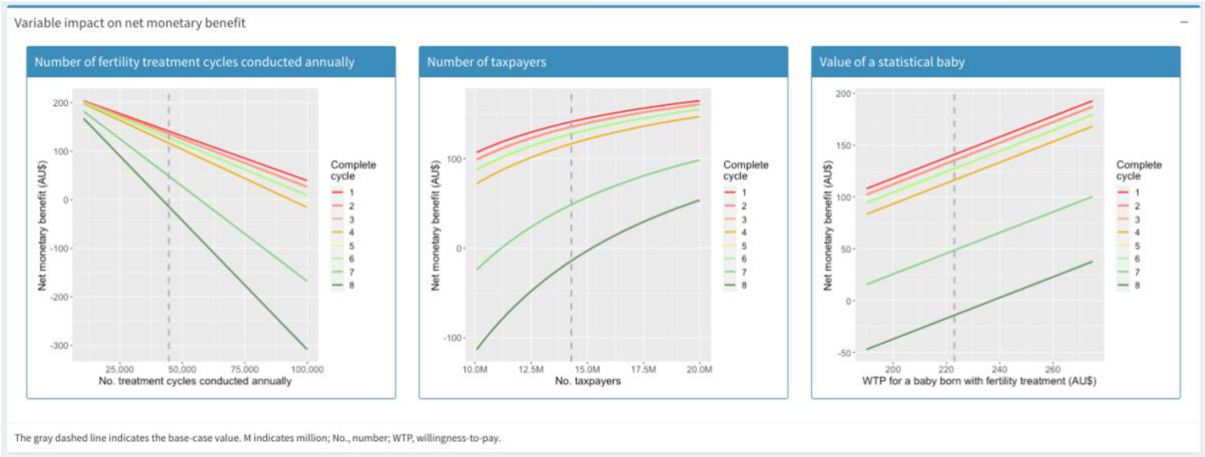


**Supplementary Figure 3.** Impact of changes in the (1) number of treatment cycles conducted annually, (2) number of taxpayers, and (3) value of a statistical baby on the net monetary benefit per complete IVF treatment cycle in 38-39-year-old women.

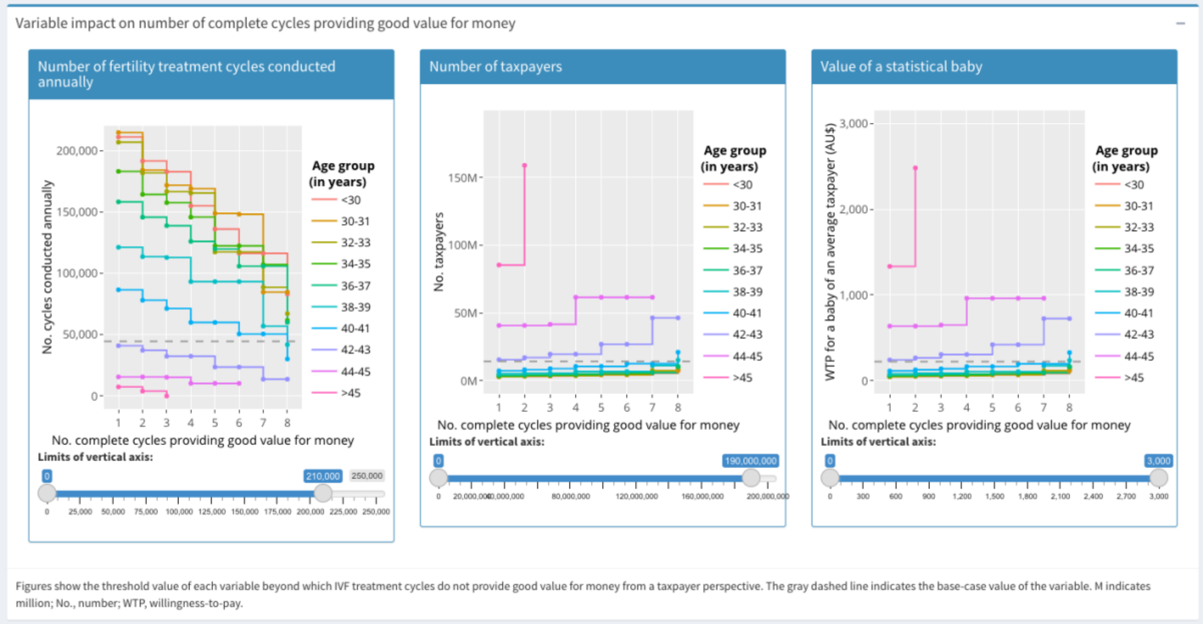


**Supplementary Figure 4.** Threshold analysis indicating the value of the (1) number of treatment cycles conducted annually, (2) number of taxpayers, and (3) value of a statistical baby beyond which a complete IVF treatment cycle does not provide good value for money from a taxpayer perspective.

Notes: The slider at the bottom allows adjusting the limits of the vertical axis to zoom in/out of the figure.

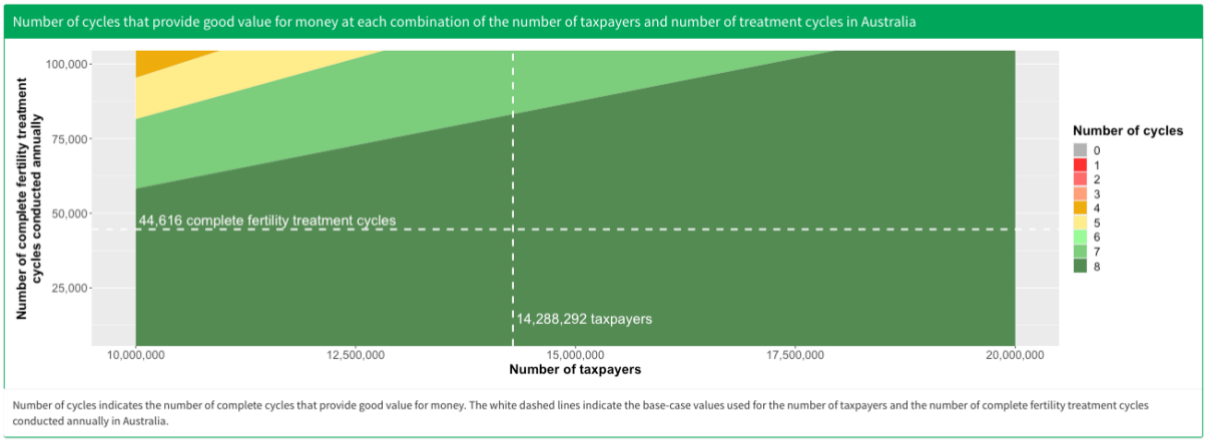


**Supplementary Figure 5.** Area chart for the two-way sensitivity analyses showing the number of complete IVF treatment cycles in 38-39-year-old women that provide good value for money at each combination of the number of taxpayers and number of treatment cycles conducted annually in Australia.

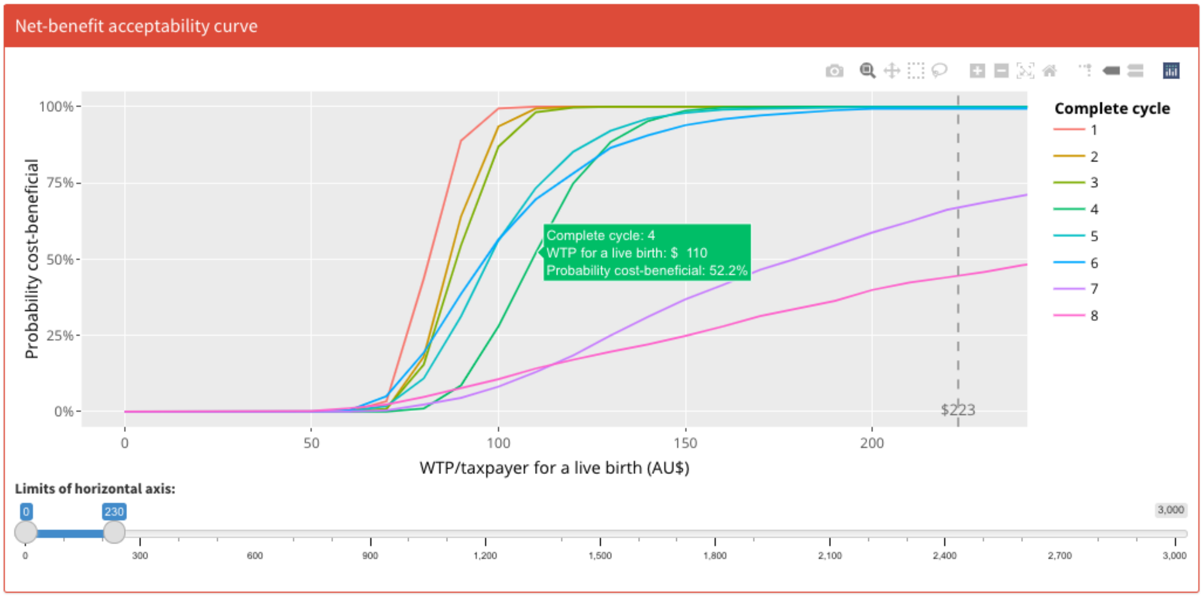


**Supplementary Figure 6.** Net-benefit acceptability curve based on the probabilistic sensitivity analyses showing the probability that a complete cycle in 38-39-year-old women is good value for money at different willingness-to-pay (WTP) values per taxpayer for a live birth (i.e., value of a statistical baby).

Notes: The slider at the bottom allows adjusting the limits of the horizontal axis to zoom in/out of the figure. When hovering the cursor above data points in the online figure, data labels appear that provide the exact values for the WTP per taxpayer for a live birth and the corresponding probability that the cycle is good value for money. As an example, the screenshot shows the data label for the 4$^{th}$ complete cycle in 38-39-year-old women at a WTP value per taxpayer for a live birth of $110.

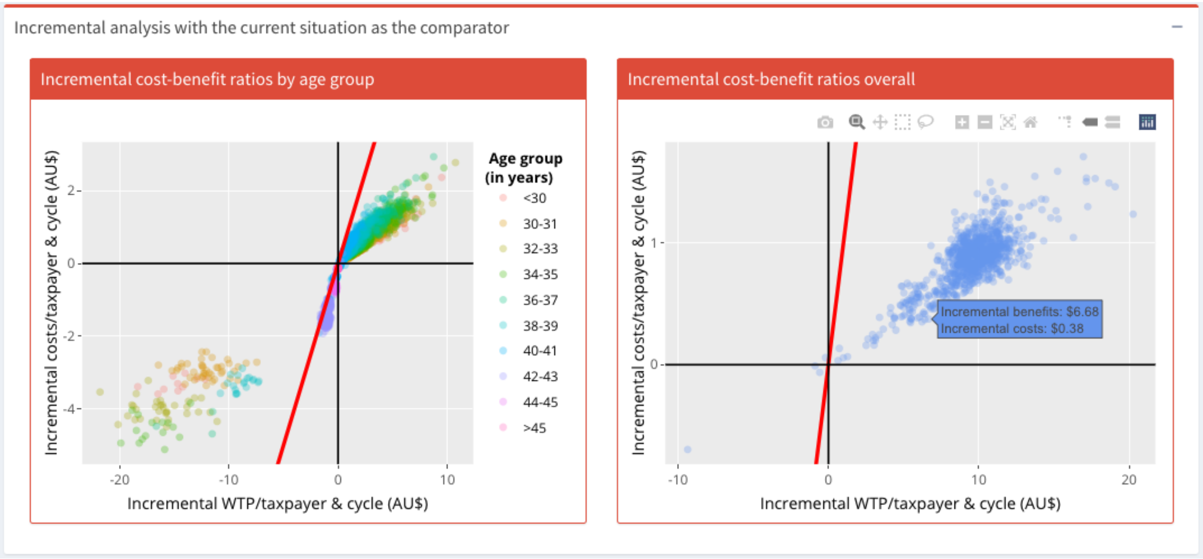


**Supplementary Figure 7.** Cost-benefit planes by age group (left panel) and overall (right panel) showing incremental cost-benefit ratios of only funding cost-beneficial cycles compared to the current funding environment in Australia, which is characterized by unrestricted funding based on age and previous treatment attempts.

Notes: When hovering the cursor above data points in the online figure, data labels appear that provide the exact values for the incremental benefits and incremental costs. As an example, the screenshot shows the data label for one iteration of the model.
